# Integrated transcriptomic and metabolomic analyses reveal key metabolic pathways in response to potassium deficiency in coconut (*Cocos nucifera* L.) seedlings

**DOI:** 10.3389/fpls.2023.1112264

**Published:** 2023-02-13

**Authors:** Lilan Lu, Siting Chen, Weibo Yang, Yi Wu, Yingying Liu, Xinxing Yin, Yaodong Yang, Yanfang Yang

**Affiliations:** ^1^ Hainan Key Laboratory of Tropical Oil Crops Biology, Coconut Research Institute, Chinese Academy of Tropical Agricultural Sciences, Wenchang, Hainan, China; ^2^ School of Earth Sciences, China University of Geosciences, Wuhan, Hubei, China; ^3^ Key Laboratory of Tree Breeding and Cultivation of State Forestry Administration, Research Institute of Forestry, Chinese Academy of Forestry, Beijing, China

**Keywords:** *Cocos nucifera L.*, transcriptome (RNA-seq), metabolome, potassium deficiency, physiology

## Abstract

Potassium ions (K^+^) are important for plant growth and crop yield. However, the effects of K^+^ deficiency on the biomass of coconut seedlings and the mechanism by which K^+^ deficiency regulates plant growth remain largely unknown. Therefore, in this study, we compared the physiological, transcriptome, and metabolite profiles of coconut seedling leaves under K^+^-deficient and K^+^-sufficient conditions using pot hydroponic experiments, RNA-sequencing, and metabolomics technologies. K^+^ deficiency stress significantly reduced the plant height, biomass, and soil and plant analyzer development value, as well as K content, soluble protein, crude fat, and soluble sugar contents of coconut seedlings. Under K^+^ deficiency, the leaf malondialdehyde content of coconut seedlings were significantly increased, whereas the proline (Pro) content was significantly reduced. Superoxide dismutase, peroxidase, and catalase activities were significantly reduced. The contents of endogenous hormones such as auxin, gibberellin, and zeatin were significantly decreased, whereas abscisic acid content was significantly increased. RNA-sequencing revealed that compared to the control, there were 1003 differentially expressed genes (DEGs) in the leaves of coconut seedlings under K^+^ deficiency. Gene Ontology analysis revealed that these DEGs were mainly related to “integral component of membrane,” “plasma membrane,” “nucleus”, “transcription factor activity,” “sequence-specific DNA binding,” and “protein kinase activity.” Kyoto Encyclopedia of Genes and Genomes pathway analysis indicated that the DEGs were mainly involved in “MAPK signaling pathway-plant,” “plant hormone signal transduction,” “starch and sucrose metabolism,” “plant-pathogen interaction,” “ABC transporters,” and “glycerophospholipid metabolism.” Metabolomic analysis showed that metabolites related to fatty acids, lipidol, amines, organic acids, amino acids, and flavonoids were generally down-regulated in coconut seedlings under K^+^ deficiency, whereas metabolites related to phenolic acids, nucleic acids, sugars, and alkaloids were mostly up-regulated. Therefore, coconut seedlings respond to K^+^ deficiency stress by regulating signal transduction pathways, primary and secondary metabolism, and plant-pathogen interaction. These results confirm the importance of K^+^ for coconut production, and provide a more in-depth understanding of the response of coconut seedlings to K^+^ deficiency and a basis for improving K^+^ utilization efficiency in coconut trees.

## Introduction

Potassium (K^+^) is a major nutrient necessary for plant growth and development, accounting for approximately 2–10% of the total dry weight of plants (Leigh and Wyn Jones, 1984). K^+^ plays important roles in enzyme activation, protein synthesis, photosynthesis, turgor, osmotic adjustment, ion homeostasis, and electric neutralization (Römheld and Kirkby, 2010; Kanai et al., 2011; Hafsi et al., 2014), and more than 60 enzymes and several cofactors play direct or indirect roles in these processes (Hawkesford et al., 2012; Vašák and Schnabl, 2016). K^+^ levels affect the levels of primary and secondary metabolites in plants (Armengaud et al., 2009; Coskun et al., 2017; Chatterjee et al., 2020). Therefore, K^+^ deficiency negatively affects osmotic pressure, nutrient balance, photosynthesis, and protein synthesis, and compromises plant growth.

The soil K^+^ content in farmlands is low in large areas of the world, and crops cannot effectively use soil mineral elements (Perry et al., 1972; Hafsi et al., 2014). Coconut trees are grown in acidic soils in southern China, where K^+^ supply is insufficient (Lu et al., 2021). Adding K^+^ fertilizer can improve crop yield, but increasing the use of K^+^ fertilizer in agriculture will lead to environmental pollution (Römheld and Kirkby, 2010). Based on K^+^ fertilizer consumption from 1961 to 2018, the global K^+^ utilization efficiency of cereal crops was estimated only 19%, emphasizing the need to protect this non-renewable natural resource (Dhillon et al., 2019). Therefore, improving crop K^+^ utilization efficiency is crucial for optimizing fertilization, increasing crop yields, and reducing environmental pollution. To optimize K^+^ utilization by specific crops, it is important to study the responses and adaptations of crops to K^+^ deficiency and the underlying mechanisms.

K^+^ deficiency is a common abiotic stress in agricultural practice (Hosseini et al., 2017; Xu et al., 2020). It increases the free sugar content in plants, such as Arabidopsis (leaves and roots) (Armengaud et al., 2009), rice (Ma et al., 2012; Chen et al., 2015), barley (Zeng et al., 2018), potato (Koch et al., 2018), soybean leaves (Cakmak et al., 1994), rape (Pan et al., 2017), beet (Aksu and Altay, 2020), alfalfa root (Jungers et al., 2019), tomato (Sung et al., 2015), and cotton (leaves) (Bednarz and Oosterhuis, 1999; Pettigrew, 1999; Hu et al., 2017). In addition, free amino acids (especially, proline) are excessively accumulated in Arabidopsis (Armengaud et al., 2009), barley (Zeng et al., 2018), tobacco (Ren et al., 2016), cotton (Hu et al., 2017), and rape (Lu et al., 2019) under K^+^ deficiency. Significant changes in the plant metabolite profile caused by K^+^ deficiency can lead to metabolic disorders (Hu et al., 2016). Under K^+^-deficient conditions, plants alter their root structure and root hairs to absorb more nutrients (Jia et al., 2008). Transcriptome analysis has been used to analyze gene maps of the main metabolic pathways in K^+^-deficient tomato, cotton, wheat, rice, and soybean roots (Ma et al., 2012; Ruan et al., 2015; Singh and Reddy, 2017; Zhao et al., 2018; Yang et al., 2021). These studies have identified transcription factors and genes involved in metabolic pathways (including those encoding carbohydrates, plant hormones, and kinases) that play indispensable roles in maintaining plant growth under K^+^ deficiency (Hyun et al., 2014; Zhao et al., 2018; Yang et al., 2021). Seedlings can regulate mineral nutrient absorption *via* the roots (Bari et al., 2006; Tabata et al., 2014; Xiong et al., 2022). High-affinity K^+^ transporters regulate K^+^ uptake by cotton roots during K^+^ deficiency (Wang et al., 2019). Therefore, it is important to analyze the adaptation of plant seedlings to K^+^ deficiency.

Coconut (*Cocos nucifera* L.) is a perennial palm tree species and a multifunctional tropical crop that is used to produce food, energy, daily necessities, and chemicals. Coconuts can be eaten fresh and are an important high-value tropical fruit and oil crop, as well as a unique renewable, green, and environment-friendly resource in tropical areas (Lu et al., 2021). Coconut trees require a large amount of K^+^ for growth and development, and coconut water is rich in K^+^; therefore, the trees have to absorb sufficient K^+^ from the soil during development. Coconut generally requires additional external organic or chemical K^+^ fertilization for optimal growth (Malhotra et al., 2017; Baghdadi et al., 2018). K^+^ deficiency causes abiotic stress in crops, limiting their yield (Zorb et al., 2014; Qin et al., 2019). Plants respond to K^+^ deficiency at the morphological, physiological, biochemical, and molecular levels (Hafsi et al., 2014). In maize, low K^+^ levels induce lateral root growth, inducing genes related to nutrient utilization, hormones, and transcription factors (Zhao et al., 2016; Ma et al., 2020). Mild and moderate K^+^ deficiency causes yellowing and scorching of mature coconut leaves, and in severe cases, new leaves start yellowing and withering, leading to weakened photosynthesis, decreased immunity, disease, and insect infestation of the leaves (Ollagnier and Shi, 1988; Maheswarappa et al., 2014). K^+^ deficiency also reduces the number of female coconuts, resulting in poor pollination and affecting the development of flowers, young fruits, and fruits (Frem. and He, 1989; Broschat, 2010); lowers the yield and quality of adult trees (Tang, 1997; Kalpana et al., 2008); and causes aging and decay of coconut tree roots (Chen, 2005; Mohandas, 2012a; Mohandas, 2012b). However, although K^+^ is absorbed *via* the roots, it is unclear how low K^+^ concentrations promote root absorption to adapt to K^+^ deficiency by regulating relevant genes and metabolites in coconut seedling leaves.

K^+^ metabolism is regulated by several genes that are differentially expressed under different K^+^ conditions. RNA-sequencing (RNA-seq) is an important tool in plant molecular research that uses high-throughput sequencing technologies to sequence cDNA libraries. RNA-seq involves reverse transcription of total RNA in tissues or cells to determine gene expression levels. Transcriptome analysis using next-generation sequencing technologies has been used to study the molecular mechanisms of plant responses to nutritional stresses (Wasaki et al., 2006; Saurabh et al, 2017) Studies have used transcriptome analysis to analyze the response of plants, including rice (Ma et al., 2012), soybean (Wang et al., 2012), sugarcane (Zeng et al., 2015), wheat (Ruan et al., 2015), soybean (Zeng et al., 2015), and wheat (Wang et al., 2019), to K^+^ stress. Transcriptome analysis improves our understanding of the complex molecular mechanisms of K^+^ uptake and transformation in plants.

Metabolomic analysis is used to detect metabolites in cells or tissues, study the synthesis of all or some metabolites, and elucidate their decomposition or transformation principles (Hall, 2011; Zhang et al., 2020). Abiotic stress can lead to metabolic disorders in plants (Meena et al., 2017). CChanges in small-molecular compounds under mineral nutrient stress have been evaluated. Sung et al. (2015) studied the metabolic response of tomato leaves and roots to nitrogen (N), phosphorus (P), and K deficiency and found that nutrient deficiency affected plant amino acid metabolism and energy production. Zhang et al. (2020) discovered that α-amino acids and their derivatives, sucrose, and sugar alcohols were significantly increased in cotton seedlings under low-K stress and reflected as damaged cell membranes and abnormal protein metabolism. K^+^ deficiency reduces the antioxidant capacity of cotton seedlings, leading to metabolic disorders, including an increase in primary metabolites and inhibition of secondary metabolite production. Watanabe et al. (2020) found that the levels of anti-stress substances and amino acid substitutes increased under N deficiency, and the metabolism of benzoic acid, erucic acid, and glucuronate in rice leaves was related to low-P stress. Gao et al. (2020) found that p-hydroxybenzoic acid, inositol, dinol, and stachyose levels increased in lettuce leaves under N deficiency.

Integrated transcriptomic and metabolomic methods are increasingly being applied to reveal molecular mechanisms of environmental stress resistance in different crops based on genetic, physiological, and morphological data (Bowne et al., 2011). This approach has led to the unraveling of the tolerance mechanism of wild soybean seedling roots to low-N stress (Liu et al., 2020), identification of candidate genes possibly involved in oat adaptation to P deficiency (Wang et al., 2018), elucidation of the response of rice carbon and N metabolism to high N (Xin et al., 2019), understanding metabolic changes caused by regulation of P utilization efficiency in rice leaves (Wasaki et al., 2003), regulation of phosphorylated metabolite metabolism in soybean roots in response to P deficiency (Mo et al., 2019), effect of N deficiency on wheat grains during the medium filling stage (Wang et al., 2021), response mechanisms of apple to different P stresses (Sun et al., 2021), regulatory mechanisms of primary and secondary metabolism of peanut roots under N deficiency stress (Yang et al., 2022), and the transcriptional and metabolic responses of maize buds to long-term K^+^ deficiency (Xiong et al., 2022).

At present, few studies have evaluated nutritional stress in palm woody plants using a combination of metabolomic with transcriptomic methods. Coconuts are very important for the development of food, household goods, energy, and industrial resources in Hainan Province, China, and the economic income level of the region. However, few studies have reported the metabolome and transcriptome data of coconuts under K^+^ stress. As a perennial tropical woody fruit tree, the level of K^+^ has a significant impact on the growth, yield, and quality of coconut trees (Mao et al., 1999; Malhotra et al., 2017). Thus, identifying the genes related to K metabolism is key to optimizing K application to coconut trees. In contrast to the model plants Arabidopsis, rice, and maize, for which extensive molecular information related to K metabolism research is available (Zhao et al., 2018; Wang et al., 2019; Yang et al., 2021), the effects of K stress and the genes and metabolites involved in coconut tree response to K stress are relativelky unknown. Thus, the identification of genes and metabolites under different K conditions will be of great significance to better understand K metabolism and the related pathways in coconut trees.

In this study, the growth and physiological conditions of coconut seedlings under different K^+^ treatments were analyzed using pot hydroponic experiments. In addition, the effects of K^+^ deficiency on coconut seedling development were studied using transcription and metabolomics. This study aimed to determine the main effects of K^+^ deficiency on coconut seedling growth and their transcript and metabolite responses. These findings will be helpful in understanding the physiological adaptability and developmental mechanisms of coconut seedlings under K^+^ deficiency and provide a theoretical basis for improving the K^+^ utilization efficiency in coconut breeding.

## Material and methods

### Plant growth and treatments

Seed fruits were removed from Huangai (Wenye No. 2) coconut seedlings that had grown for 1.5 months after the seed fruit had sprouted and the seedlings were recovered in a nutrition bag for 15 days. Seedlings with similar size, height, and number of leaves were transferred to a hydroponic system in a substrate of non-nutritive quartz sand and vermiculite (20:6) (50 × 37 × 35 cm). The coconut seedlings were randomly divided into three treatment groups (60 seedlings per treatment), with three biological replicates per treatment and 20 seedlings per replicate. After 10 days of preculture, K^+^ deficiency stress treatment was initiated. The K content of the coconut seedlings was determined based on the standard K content of coconut leaves (Brunin and He, 1965). The seedlings were treated with 0.1 mM KCl (K^+^ deficiency [K_0_]) or 4 mM KCl (K^+^ sufficiency [K_ck_]); solution concentrations of other nutrients were in accordance with those reported by Hoagland and Arnon (1950). All nutrient solutions were irrigated every 3 days for 30 days, after which the seedling responses were evaluated. Plant height was recorded and the plants were sampled. Part of the leaves were immediately frozen in liquid nitrogen and stored at –80°C until further physiological, nutrient, metabolic, and transcriptional analyses.

### Measurements of plant height, dry weight, and soil and plant analyzer development values

Plant height was measured using a measuring tape (accuracy: 1 mm). The SPAD value of the coconut leaves was measured using a SPAD chlorophyll meter (SPAD-502 Plus, Konica Minolta, Tokyo, Japan). To evaluate the dry weight of coconut seedlings, the fresh stems, leaves, and roots of coconut seedlings were dried at 105°C for 15 min and at 70°C for 72 h and then weighed using an electronic balance (Labpro, Shanghai, China). The dry weight (stems + leaves + roots) of the whole plant was calculated.

### Determination of N, P, and K contents

Briefly, 200 mg dried and finely ground coconut leaf sample was transferred into a 100 mL digestion tube, to which, 5 mL H_2_SO_4_ and 5 mL HClO_4_ were added, and the mixture was shaken gently. Then, a curved neck funnel was placed at the mouth of the bottle and the bottle was heated until the digestion solution was colorless or clear, then heated for another 5–10 min, and allowed to cool down. The digestion solution was then transferred into a 100-mL constant volume bottle, diluted with deionized water, and filtered. The filtrate was used to measure N, P, and K contents. For the determination of N, 5 mL filtrate was transferred to a 50-mL volumetric flask, to which 2 mL of 100 g·L^–1^ sodium tartrate solution was added. This was followed by the addition of 100 g·L^–1^ KOH solution for acid neutralization, deionized water was added to make up the volume to 40 mL, and then, 2.5 mL Nessler’s reagent was added to the mixture. The mixture was diluted with deionized water and shaken well. In addition, we prepared N (NH_4_^+^-N) standard solutions (2, 5, 10, 20, 40, 60 µg.mL^–1^) by adding 5 mL blank digestion solution. Color development in the sample and standard solutions was measured at 420 nm using a UV-visible spectrophotometer (UV-1600, Aoyi, Shanghai, China). The measurement for the blank digestion solution was set as the zero point for the instrument. For the determination of P, 5 mL digestion filtrate was transferred into a 50-mL volumetric flask and diluted to 30 mL with deionized water. Then, two drops of dinitrophenol indicator were added, followed by the addition of 4 mol·L^–1^ NaOH solution until the solution turned yellow; subsequently, one drop of 2 mol·L^–1^ (1/2 H_2_SO_4_) was added so that the yellow color of the solution faded. Then, 5 mL molybdenum-antimony reagent was added to the solution, followed by the addition of 50 mL deionized water. P standard solutions (0, 0.1, 0.2, 0.4, 0.6, 0.8, 1.0 µg·mL^–1^) were prepared by adding 5 mL blank digestion solution, using the same procedure. Color development in the sample and standard solutions was measured at 880 nm using the UV-visible spectrophotometer, and the absorbance of the blank solution was set to 0. For the determination of K, 5 mL digestion filtrate was transferred into a 50-mL volumetric flask, and the volume was adjusted using deionized water. K standard solutions (2, 5, 10, 20, 40, 60 µg·mL^–1^) were prepared by adding 5 mL blank digestion solution, and the sample and standard solutions were analyzed using an atomic absorption spectrophotometer (AA6300F, Shimadzu, Kyoto, Japan), according to the method reported by Bao (2000).

### Determination of soluble sugar, soluble protein, crude fat, endogenous hormone, proline, and malondialdehyde contents and enzyme activities

For CF detection, CF was extracted from fresh leaf tissues using a distillation device. For the determination of auxin (IAA), gibberellin (GA), abscisic acid (ABA), zeatin (ZR), SP, SS, MDA, and Pro contents and superoxide dismutase (SOD), catalase (CAT), and peroxidase (POD) activities, 0.1000 g coconut leaf tissue was accurately weighted and mixed with precooled PBS at a weight (g) to volume (mL) ratio of 1:10. The samples were subjected to high-speed grinding and centrifuged at 2500 rpm for 10 min. Then, 50 µL supernatant was used for the measurements. IAA, GA, ABA, ZR, MDA, SP, Pro, SOD, CAT, and POD kits and standards were obtained from the Nanjing Jiancheng Bioengineering Research Institute, and the measurements were performed in strict accordance to the manufacturer’s instructions and following the method reported by Li (2000). We used 1-cm optical path cuvettes, and blank cuvette was used for setting the baseline. The wavelength was set to 450 nm (IAA, ABA, GA, ZR), 595 nm (SP), 620 nm (SS), 532 nm (MDA), 520 nm (Pro), 550 nm (SOD), 405 nm (CAT), or 420 nm (POD) to measure the absorbance by using the enzyme marker (DG5033A, Nanjing Huadong Electronics Group Medical Equipment). All measurements were carried out within 10 min after adding the termination solution. Based on the absorbance value, the concentration/activity was calculated according to the manufacturer’s formulas.

### RNA extraction and RNA-seq

Total RNA was extracted from the frozen samples using the improved cetyltrimethylammonium bromide (CTAB) method. RNA purity and integrity were visually evaluated by agarose gel electrophoresis. The RNA concentration was measured using a NanoDrop 2000 spectrophotometer (Thermo Fisher Scientific, Waltham, MA, USA). An Agilent 2100 Bioanalyzer system (Agilent Technologies, Palo Alto, CA, USA) was used to quantify RNA integrity. Library assembly and RNA-seq analysis were performed at Beijing Biomarker Biotechnology Company (Beijing, China) and Beijing Biomarker Cloud Technology Company (Beijing, China). RNA-seq libraries were generated using the NEBNext ^®^ Ultra ™ II RNA Library Prep Kit for Illumina ^®^ (New England Biolabs, Ipswich, MA, USA), and index codes were added to each sample. The libraries were sequenced on an Illumina ^®^ HiSeq2500 platform (Illumina, San Diego, CA, USA). Each sample was sequenced in triplicate. The raw reads were filtered by removing low-quality reads and adapters. The clean reads were mapped to the reference coconut genome (Xiao et al., 2017) (http://creativecommons.org/licenses/by/4.0/ ) using the transcript splicing-aligned hierarchical index (HISAT 2) program (Kim et al., 2015). Gene functions were annotated using the following databases: NCBI non-redundant protein sequences (Nr), Clusters of Orthologous Groups of proteins (COG/KOG), Swiss-PROT protein sequence database, Kyoto Encyclopedia of Genes and Genomes (KEGG), Homologous protein family (Pfam), and Gene Ontology (GO) (Tatusov et al., 2000; Finn et al., 2013). For each transcript region, the RESM software was used to calculate the Fragments per kilobase of transcript per million fragments mapped (FPKM) (Li and Dewey, 2011). The DESeq software was used to analyze differential gene expression among samples, and the Benjamini–Hochberg method was used to determine significance. DEGs were defined based on |fold change (FC)| ≥ 1.5 and *P* < 0.05 (Love et al., 2014). The GOseq R software package was used for GO term enrichment analysis of the DEGs (Ashburner et al., 2000; Alexa and Rahnenfuhrer, 2010; Robinson et al., 2010). The KEGG Orthology Based annotation system (KOBAS) software was used for KEGG pathway enrichment analysis of the DEGs (Kanehisa et al., 2004).

### Metabolite analysis

Sample preparation and metabolome and data analyses were conducted by Beijing Biomarker Biotechnology Co., Ltd. (http://www.biomarker.com.cn/ ). Briefly, the frozen coconut leaves were grounded into a powder in liquid N and 100 mg powder was added to a 1.5 mL Eppendorf tube. The samples were extracted with 1.0 mL of 70% aqueous methanol solution at 4°C for 24 h and centrifuged at 10000×*g* and 4°C for 10 min. The extracts were filtered through 0.22-µm nylon membranes and subjected to liquid chromatography-mass spectrometry (LC-MS) analysis. Quality control samples were prepared by extracting and mixing three duplicate samples from each K treatment. During analysis, each quality control sample was measured together with the corresponding three experimental samples to check the stability of the analysis conditions.

An ultraperformance LC-electrospray ionization MS (UPLC-ESI-MS/MS) system (Shimadzu) was used to analyze the metabolic spectra of the leaf extracts (10 µL). Chromatographic separation in water was performed on a UPLC HSS T3 C18 column (2.1 mm × 100 mm, i.d., 1.8 µm) (Waters, Milford, MA, USA) at 40°C. The mobile phase consisted of water containing 0.04% acetic acid (mobile phase A), and acetonitrile containing 0.04% acetic acid (mobile phase B). The linear gradient program for elution was set as follows: 0–11.0 min from 5% to 95% B, 11.0–12.0 min from 95% to 5%, and 12.1–15.0 min from 5% to 5%. The flow rate of the mobile phase was 0.40 mL/min.

An API 4500 QTRAP LC-MS/MS system (AB SCIEX, Framingham, MA, USA) was used for MS and MS/MS analysis. The ESI source parameters were as follows: turbine spray, ion source, source temperature: 550°C; ion spray voltage: 5.5 kV; air curtain gas pressure: 25 pounds per square inch (psi), ion source gas 1 pressure: 55 psi, and gas II pressure: 60 psi. A multiple reaction monitoring experiment was conducted with a 5-psi nitrogen collision gas to obtain the quadrupole scanning results.

Metabolites were identified based on a public database of metabolite information and the cloud technology database of Beijing Biomarker Biotechnology Co., Ltd. (Beijing, China). Open databases, including HMDB, MoToDB, MassBank, METLIN, and KNAPSAcK, were used for qualitative analysis of the metabolites identified by the MS. Metabolite structures were analyzed using standard metabolic procedures. The metabolites were quantified using multiple reaction monitoring. All metabolites identified were analyzed using partial least squares discriminant analysis (PLS-DA). Principal component analysis (PCA) and orthogonal PLS-DA (OPLS-DA) were used to identify potential biomarkers. For biomarker selection, variable importance of projection (VIP) ≥1 and folding change (FC) ≥ 2 or ≤ 0.5 were used as criteria to screen for significantly differentially accumulated metabolites (DAMs).

### Integrated metabolome and transcriptome analyses

Pearson correlation coefficients (R^2^) between the metabolome and transcriptome data were calculated. Specifically, the coefficients correlation between log_2_(FC) for each metabolite and log_2_(FC) for each transcript were calculated using the Excel program. Items with R^2^ > 0.8 were selected. Cytoscape (version 2.8.2) was used to visualize the relationship between the metabolome and the transcriptome (Shannon et al., 2003).

### Quantitative real-time PCR analysis

RT-qPCR was used to verify the DEGs in coconut seedlings identified by RNA-seq. Gene-specific RT-qPCR primers were designed (**Supplementary Table 1**). qPCRs were run on a LightCycler^®^ 480II Real-Time system (Roche, Carlsbad, CA, USA) in 96-well plates, using Hieff qPCR SYBR Green Master Mix (Not-Rox) (Yeasen Biotech, Shanghai, China) according to the manufacturer’s instructions. The thermal cycle steps included denaturation at 95°C for 5 min, followed by 40 cycles at 95°C for 10 s and 60°C for 30 s. All RT-qPCR analyses were performed using three technical and three biological replicates. An internal reference gene (β-actin) was used for normalization. The 2^–ΔΔCT^ method (Livak and Schmittgen, 2001) was used to calculate differential target gene expression in reference to the levels in the control group.

### Statistical analysis

All experiments consisted of three replicates (n = 3). Data are expressed as the mean ± standard deviation (SD) of the three replicates. The data were analyzed by one-way analysis of variance (ANOVA). The SPSS software (version 20.0; SPSS, Chicago, IL, USA) was used for statistical analysis. Results were *P* < 0.05 considered statistically significant. Charts and data were prepared using Excel 2003.

## Results

### Effects of K^+^ deficiency on plant growth and nutrient, SP, SS, and CF contents of coconut seedlings

**Figure 1:**
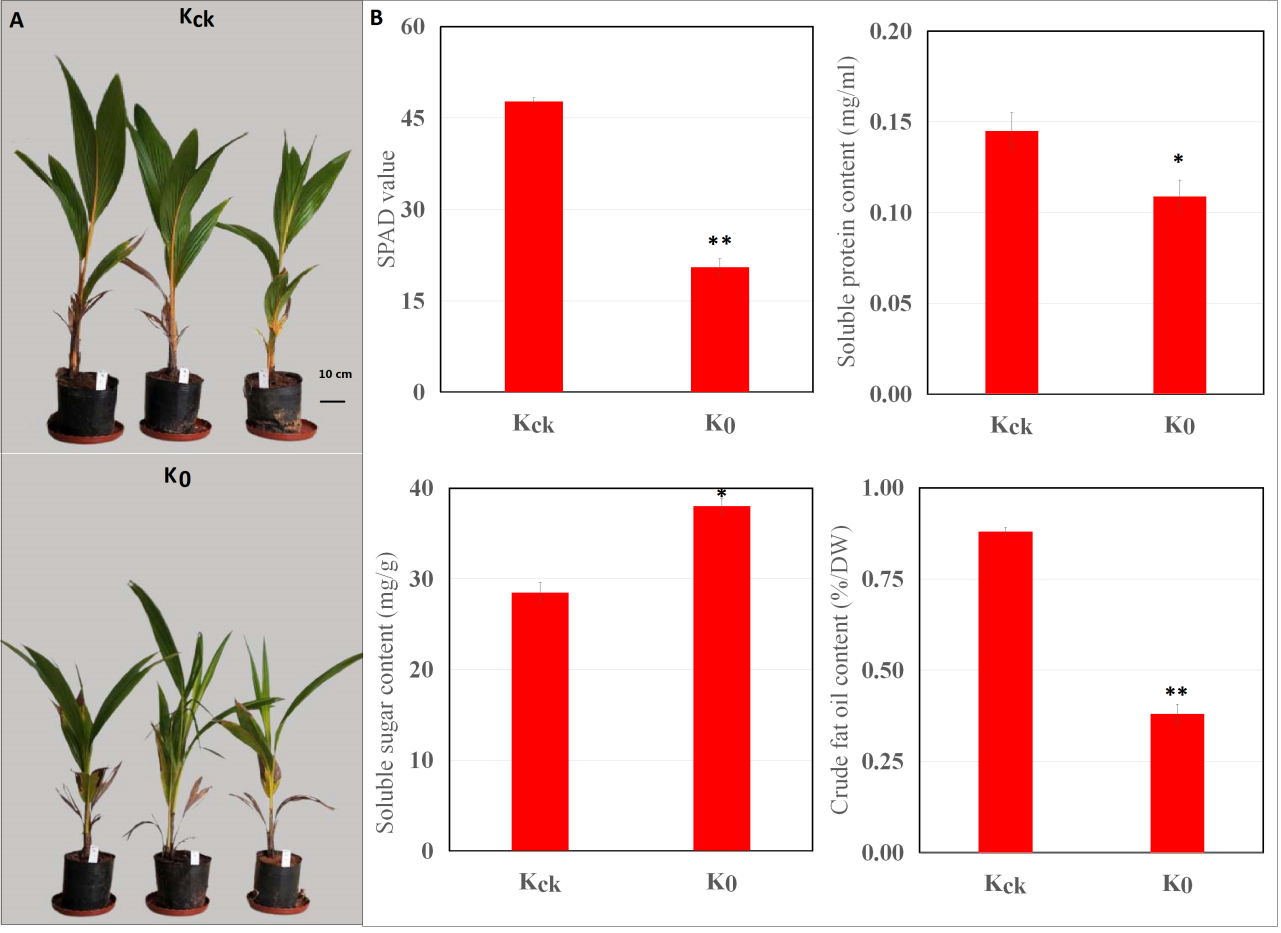
Effects of K^+^ deficiency on coconut seedlings. **(A)** Phenotype of coconut seedlings after 30 days of K^+^ treatment. **(B)** Changes in SPAD, SP, SS, and CF contents under K^+^ deficiency for 30 consecutive days. *P ≤ 0.05, **P ≤ 0.01. Bars represent SDs.

First, we evaluated the effect of K^+^ deficiency on the growth of coconut seedlings using pot experiments. The seedlings were exposed to K^+^ deficiency (0 mM, K_0_) or K^+^ sufficiency (4 mM, K_ck_). The results showed that K^+^ deficiency significantly (*P* < 0.05) reduced the plant height and dry weight of coconut seedlings by 21.83% and 26.45%, respectively (**Supplementary Table 2**). In addition, under K^+^-deficient conditions, the SP and CF contents decreased by 24.83% and 56.82% (*P* < 0.05), respectively, whereas the SS content increased by 33.43% (**Figure 1**). Under K^+^-deficient conditions, the leaf K, N, and P contents of coconut seedlings decreased; the K concentration decreased by 48.36% (**Supplementary Table 3**) and the SPAD value was significantly lower than that in control seedlings (K_ck_) (*P* < 0.01) (**Figure 1**). Therefore, K^+^ deficiency seriously affected the biomass, mineral nutrients, photosynthetic indices, and quality of coconut seedlings.

### Effects of K^+^ deficiency on SPAD values, MDA and pro contents, enzyme activities, and hormone contents

**Figure 2:**
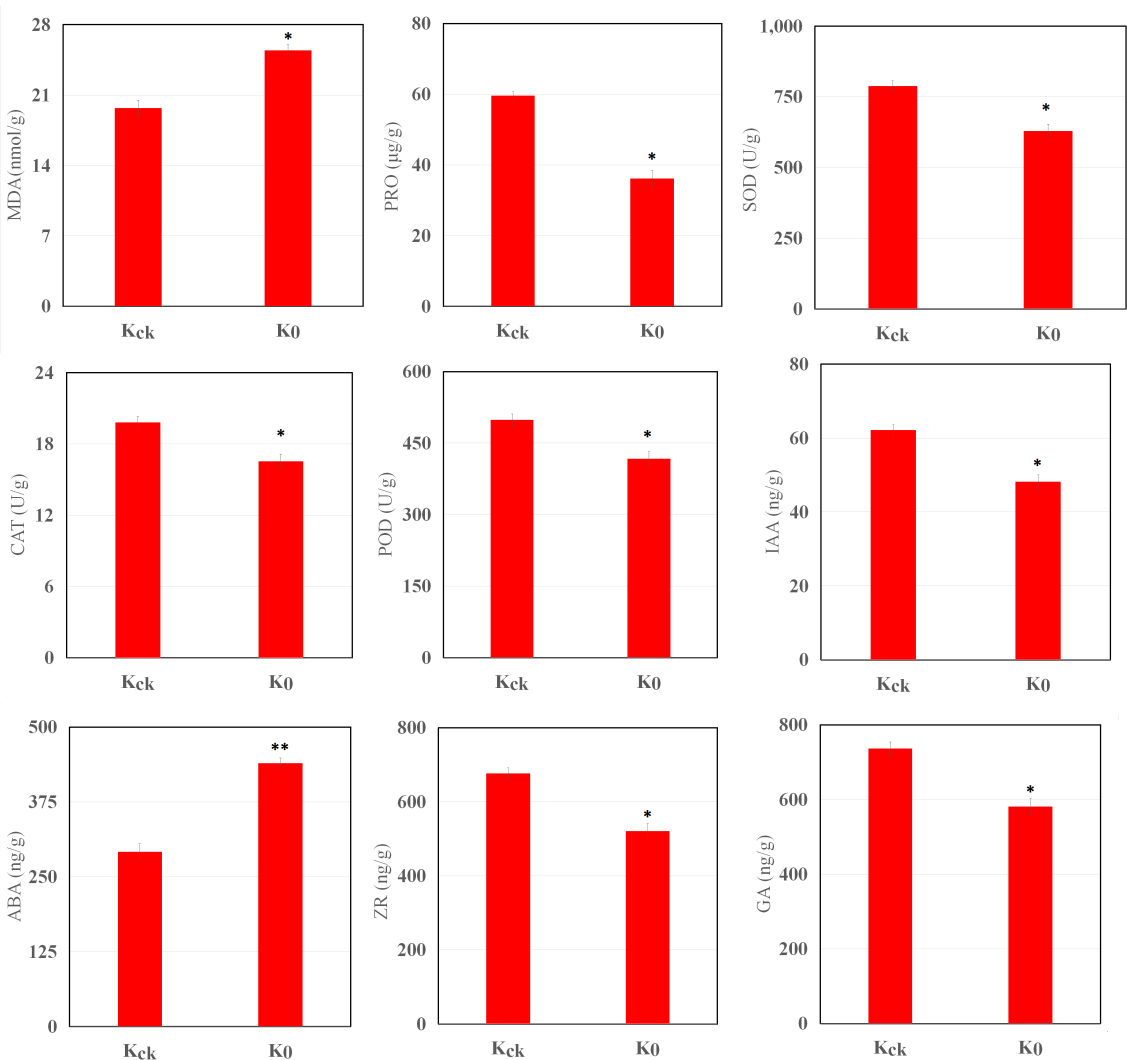
MDA, Pro, IAA, GA, ABA, and ZR contents and SOD, CAT, and POD activities in K_0_ vs. K_ck_. *P ≤ 0.05, **P ≤ 0.01. Bars represent SDs.

K^+^ deficiency stress had a significant impact on the physiology of the coconut seedlings (**Figure 2**). Under K^+^ deficiency stress, the MDA content increased by 29.11%, whereas the Pro content decreased by 39.41% (*P* < 0.05). Compared with K_ck_ seedlings, in K_0_ seedlings, CAT, POD, and SOD activities in the leaves were significantly reduced by 16.44%, 16.34%, and 20.16%, respectively (*P* < 0.05). In addition, K^+^ deficiency stress had a significant impact on endogenous hormone levels in coconut seedling leaves; the ABA content increased by 50.68% (*P* < 0.05), whereas IAA, ZR, and GA contents decreased by 22.56%, 23%, and 21.13% (*P* < 0.05), respectively (**Figure 2**). Therefore, K^+^ deficiency seriously affected the physiological function indices and hormone levels of the coconut seedlings.

### Transcriptome response to K^+^ deficiency

A summary of the RNA-seq data is presented in **Supplementary Table 4**. The FPKM values were higher in K_ck_ than in K_0_ samples, and an FPKM > 1 was used as a threshold to determine gene expression (**Supplementary Figure 1A**). The PCA results showed that K_ck_ and K_0_ samples clustered separately, suggesting significant differences in gene expression between the sample groups. Repeat samples under the K_ck_ and K_0_ conditions did not strictly cluster together, indicating that differences occurred between different repetitions (**Supplementary Figure 1B**). A volcano plot showing the significantly up-regulated and down-regulated DEGs between the two groups is shown in **Supplementary Figure 1C**.

RNA-seq detected 20910 genes with appropriate FPKM values (**Supplementary Table 5**). Under K^+^ deficiency, 1003 genes were differentially expressed in coconut seedling leaves (at thresholds of |FC| ≥ 1.5 and *P* < 0.01), including 284 up-regulated DEGs and 719 down-regulated DEGs (**Supplementary Figure 1C**; **Supplementary Table 5**). In total, 884 DEGs were functionally annotated, including 249 up-regulated and 635 down-regulated DEGs (**Supplementary Table 6**). In total, 733 DEGs (**Supplementary Figure 2**) were assigned GO terms, and GO functional enrichment analysis yielded 55 significantly enriched GO terms (FDR [error detection rate] limited standard ≤ 0.05; DEG No. ≥ 3), including 21 in the biological process (BP) category, 18 in the cellular component (CC) category, and 16 in the molecular function (MF) category (**Supplementary Figure 2**, **Supplementary Table 7**).

**Figure 3:**
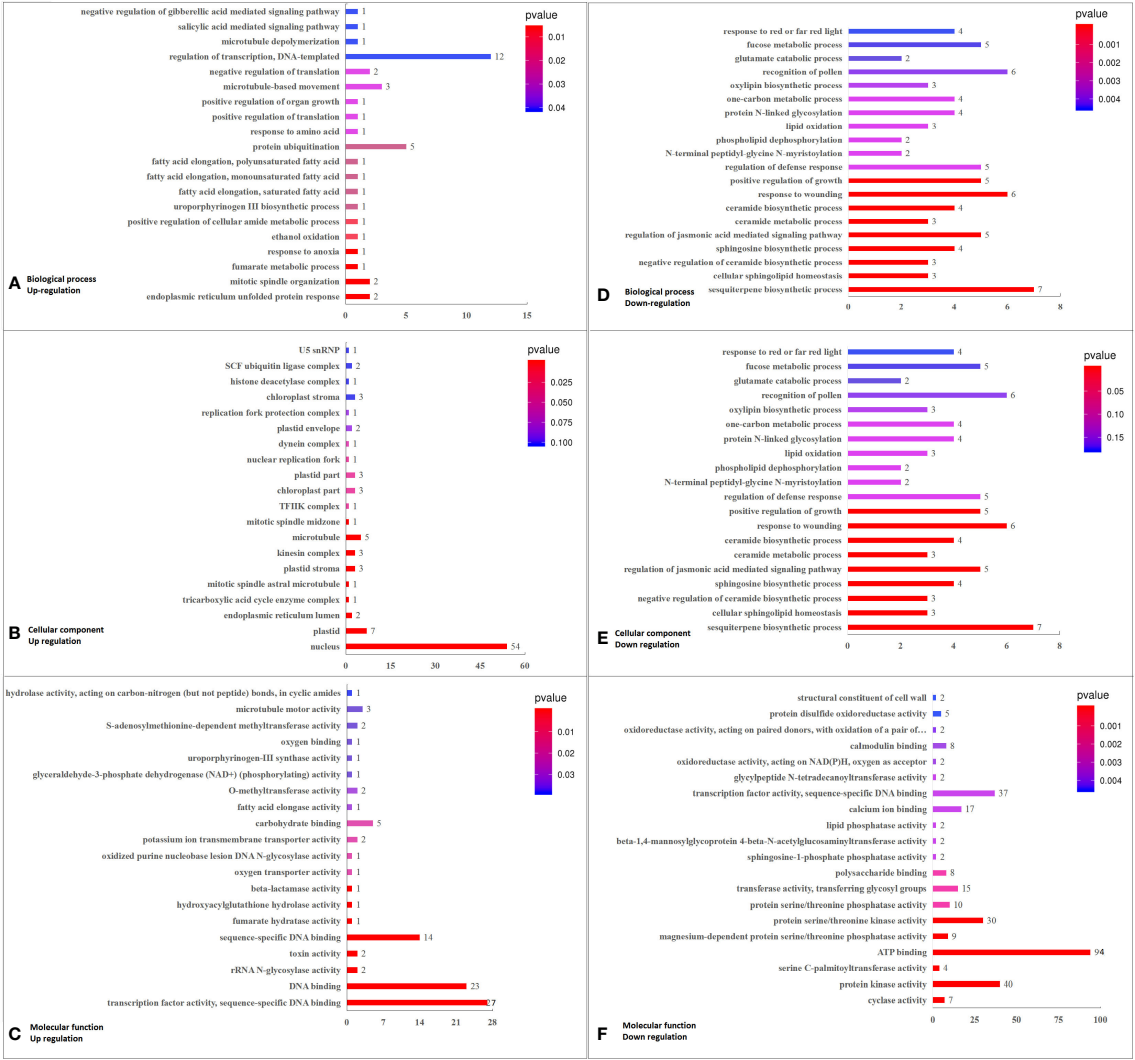
Top 20 GO pathways enriched in DEGs in K_0_ vs. K_ck_, including the three categories of BP, CC, and MF. **(A)** BP terms enriched in up-regulated DEGs. **(B)** CC terms enriched in up-regulated DEGs. **(C)** MF enriched in up-regulated DEGs. **(D)** BP terms enriched in down-regulated DEGs. **(E)** CC terms enriched in down-regulated DEGs. **(F)** MF terms enriched in down-regulated DEGs. The X-axis (GeneNum) represents the number of genes of interest annotated in the entry, the Y-axis indicates each term entry. The color of the column represents the *P* -value of the hypergeometric test.

Among the top 20 GO enriched terms, in BP, the major DEGs down-regulated under K^+^ stress were related to “sesquiterpene biosynthetic process”, “cellular sphingolipid homeostasis”, “negative regulation of ceramide biosynthetic process”, “sphingosine biosynthetic process”, “regulation of jasmonic acid mediated signaling pathway”, and “ceramide metabolic process”. In terms of CC, the down-regulated DEGs were mainly related to “integral component of membrane,” “serine C-palmitoyltransferase complex,” “SPOTS complex,” and “plasma membrane”. As for MF, the major down-regulated DEGs were involved in “cyclase activity”, “protein kinase activity”, “serine C-palmitoyltransferase activity”, “ATP binding”, “magnesium-dependent protein serine/threonine phosphatase activity”, “protein serine/threonine kinase activity”, “protein serine/threonine phosphatase activity”, “calcium ion binding”, “sphingosine-1-phosphate phosphatase activity”, “transferase activity, transferring glycosyl groups”, and “polysaccharide binding”.The up-regulated DEGs were mainly related to “nucleus”, “transcription factor activity, sequence-specific DNA binding”, “sequence-specific DNA binding”, and “DNA binding” (**Figure 3**; **Supplementary Figure 3**, **Supplementary Table 7A**).

According to analysis of DEGs enriched in the top 20 GO terms (*P* < 0.05; q < 0.05, DEG No. ≥ 3), in BP, DEGs related to sesquiterpene biosynthetic process (7), cellular sphingolipid homeostasis (3), negative regulation of ceramide biosynthetic process (3), sphingosine biosynthetic process (4), regulation of jasmonic acid-mediated signaling pathway (5), and ceramide metabolic process (3) enrichment pathways were down-regulated. Among the six GO terms enriched, the significantly down-regulated DEGs were LOC105044768 (log2FC = –1.93), ORM1 (log2FC = –2.50), Os02g0806900 (log2FC = –2.23), and TIFY9 (log2FC = –1.64). In terms of CC, 236 DEGs were related to the integral component of membrane, and 198 of these were down-regulated, including RBOHC (log2FC = –2.03), CUT1 (log2FC = –2.10), URGT2 (log2FC = –2.23), At4g17280 (log2FC = –2.05), ORM1 (log2FC = –2.50), PXC3 (log2FC = –2.08 to –2.16)), WAK2 (log2FC = –2.08), ABCG39 (log2FC = –2.01), GALT6 (log2FC = –2.35), LRK10 (log2FC = –2.00), LECRK91 (log2FC = –2.48), and EIX2 (log2FC = –1.95). Among the 38 up-regulated DEGs in CC, CML46 (log2FC = 2.23) was significant. Three down-regulated DEGs were related to the serine C-palmitoyltransferase complex, and Os11g0516000 (log2FC = –1.78) was the significantly down-regulated DEG. Three down-regulated DEGs were related to the SPOTS complex, and ORM1 (log2FC = –2.50) was the significantly down-regulated DEG. Sixty-eight DEGs were associated with the plasma membrane, of which 58 were down-regulated and 10 were up-regulated, with the most significantly down-regulated DEGs being ABCG39 (log2FC = –2.01), NSL1 (log2FC = –2.16), and LECRK91 (log2FC = –2.48). Furthermore, 139 DEGs were related to the nucleus, of which 85 were down-regulated and 54 were up-regulated; the significantly down-regulated DEGs were WRKY41 (log2FC = –2.19 to –2.52), WRKY55 (log2FC = –2.22), and ERF026 (log2FC = –2.27 to –2.92). In MF, eight down-regulated DEGs were related to cyclase activity, and LOC105044768 (log2FC = –1.93) was the most significantly down-regulated DEG. Sixty-five DEGs were associated with transcription factors or sequence-specific DNA binding activity, 38 of which were down-regulated; significantly down-regulated DEGs included WRKY41 (log2FC = –2.19 to –2.52), WRKY55 (log2FC = –2.22), and ERF026 (log2FC = –2.27 to –2.92). Forty-nine DEGs were associated with protein kinase activity, of which 43 were down-regulated, with the most significantly down-regulated DEGs being PXC3 (log2FC = –2.08 to –2.16)), WAK2 (log2FC = –2.08), IBS1 (log2FC = –2.14), and LRK10 (log2FC = –2.00). Four down-regulated DEGs were related to serine C-palmitoyltransferase activity, with Os02g06900 (log2FC = –2.23) being the most significantly down-regulated. Eleven DEGs were associated with magnesium-dependent serine/threonine phosphatase activity, nine of which were down-regulated and PLL5 (log2FC = –2.45) was the most significantly down-regulated DEG. Twelve DEGs were associated with protein serine/threonine phosphatase activity. Among them, 10 DEGs were down-regulated, with the most significant one being PLL5 (log2FC = –2.45). A total of 115 DEGs were related to ATP binding, 94 of which were down-regulated; significantly down-regulated DEGs included HSP70-4 (log2FC = –2.09), PXC3 (log2FC = –2.08 to –2.16), WAK2 (log2FC = –2.08), ABCG39 (log2FC = –2.01), IBS1 (log2FC = –2.14), LRK10 (log2FC = –2.00), and LECRK91 (log2FC = –2.48). Forty-seven DEGs were associated with protein serine/threonine kinase activity, of which 39 were down-regulated, and the most significantly down-regulated DEG was LECRK91 (log2FC = –2.48). Seventy-three DEGs were associated with sequence-specific DNA binding, of which 43 were down-regulated, and the significantly down-regulated DEGs were WRKY41 (log2FC = –2.19 to –2.52), WRKY55 (log2FC = –2.22), and ERF026 (log2FC = –2.27 to – 2.92). Twenty-two DEGs were related to calcium ion binding, of which 17 were down-regulated (RBOHC [log2FC = –2.03] and WAK2 [log2FC = –2.08] being significantly down-regulated) and five were up-regulated (CML46 [log2FC = 2.23] being the most significant one). Sixteen DEGs were related to transferase activity, transferring glycosyl groups; of these, 15 were down-regulated, and GALT6 (log2FC = –2.35) was the most significantly down-regulated DEG. Eight DEGs were related to polysaccharide binding, and WAK2 (log2FC = –2.08) was the significantly down-regulated DEG (**Supplementary Figure 3**, **Supplementary Table 7B**).

**Figure 4:**
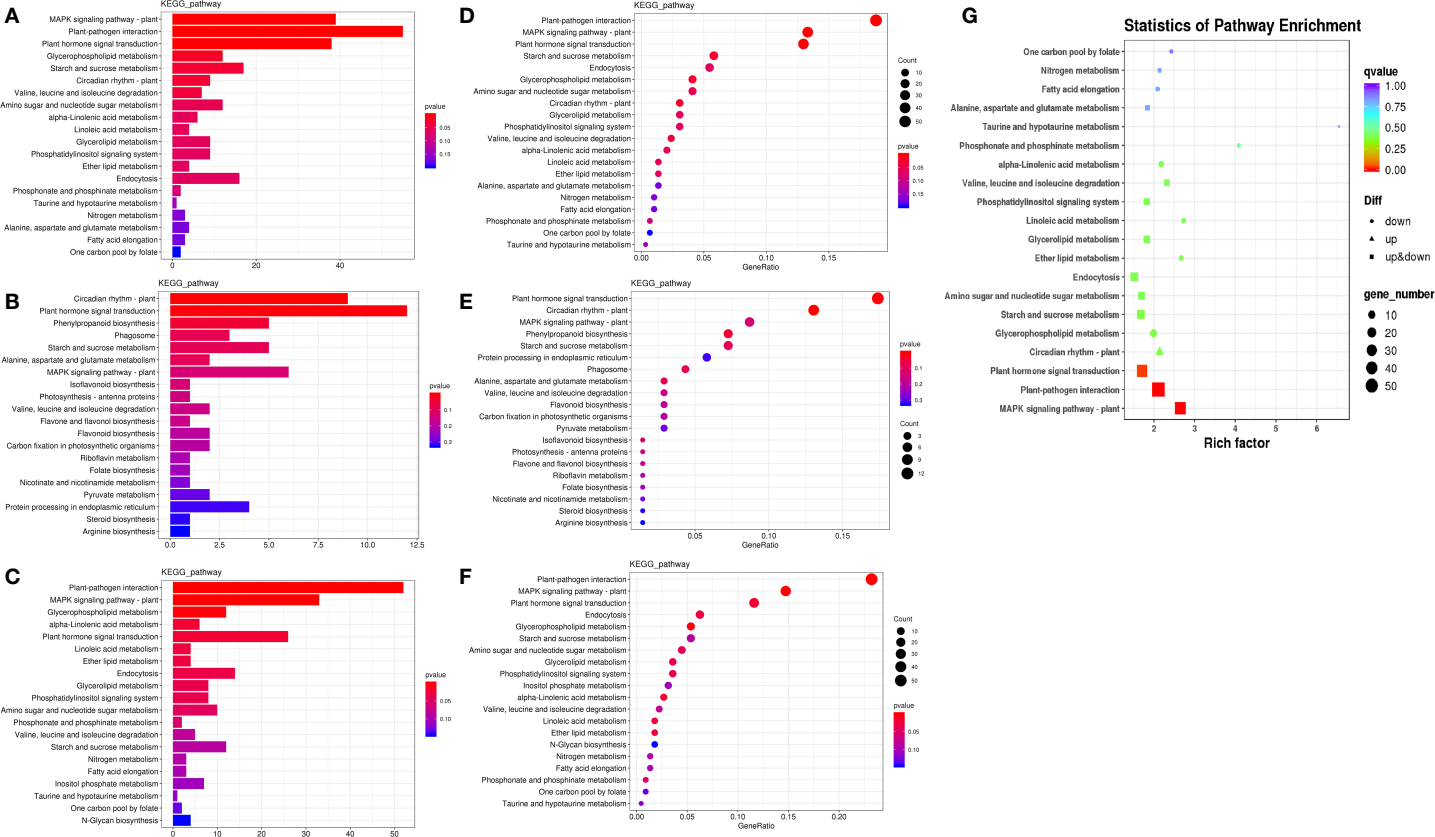
KEGG pathway enrichment of DEGs in K_0_ vs. K_ck_. **(A)** Top 20 KEGG pathways enriched in DEGs in K_0_ vs. K_ck_. **(B)** Top 20 KEGG pathways enriched in up-regulated DEGs in K_0_ vs. K_ck._ **(C)** Top 20 KEGG pathways enriched in down-regulated DEGs in K_0_ vs. K_ck_. The X-axis (GeneNum) represents the number of genes of interest annotated in the entry, the Y-axis indicates each pathway entry. The color of the column represents the *P*-value of the hypergeometric test. **(D)** Top 20 KEGG pathway enrichment bubble chart of all DEGs in K_0_ vs. K_ck_. **(E)** Bubble chart of the top 20 KEGG pathways enriched up-regulated DEGs in K_0_ vs. K_ck_. **(F)** Bubble chart of the top 20 KEGG pathways enriched in down-regulated DEGs in K_0_ vs. K_ck_. The X-axis (GeneRatio) represents the proportion of the gene of interest annotated in the entry to the number of all DEGs, the Y-axis indicates each pathway entry. Dot size represents the number of DEGs annotated in the pathway, and dot color represents the *P*-value of the hypergeometric test. **(G)** Scatter plot of top 20 KEGG pathway enrichment of all DEGs in K_0_ vs. K_ck_. Each circle represents a KEGG pathway, the Y-axis indicates the name of the pathway, and the X-axis represents the enrichment factor, which is the ratio of DEGs annotated to a certain pathway to total genes annotated to the pathway. The color of the circle represents the q-value, which is the *P*-value after multiple hypothesis test correction.

**Figure 5:**
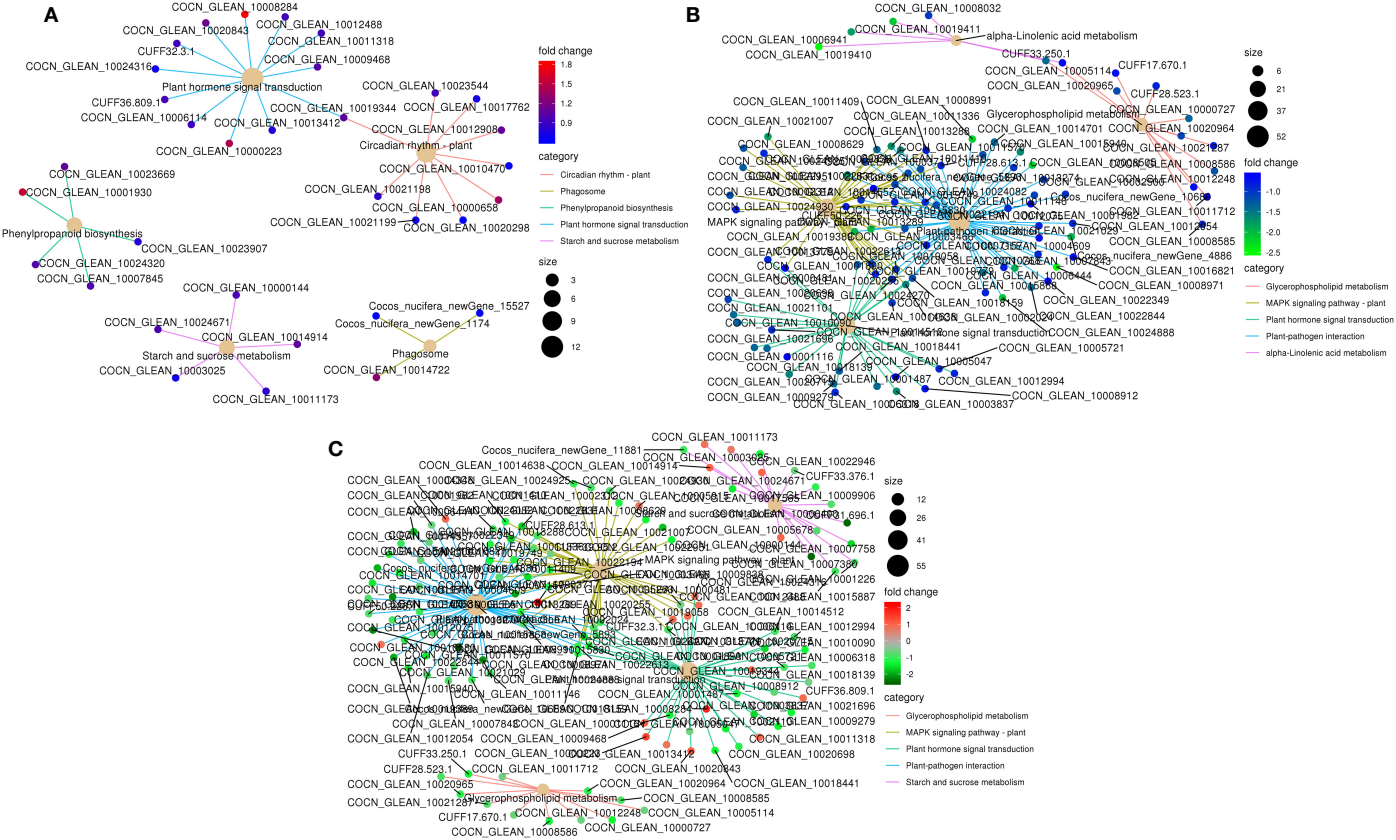
KEGG pathway enrichment network of DEGs in the top five enriched pathways in K_0_ vs. K_ck_. **(A)** up-regulated DEGs, **(B)** down-regulated DEGs, and **(C)** all DEGs. Line colors represent different pathways, and gene node colors represent multiple differences.

To further understand the physiological processes of coconut seedling growth under K^+^ deficiency, we mapped the DEGs to KEGG metabolic pathways. Among the 884 DEGs, 293 DEGs were allocated to 92 KEGG pathways; 69 up-regulated genes were assigned to 49 KEGG pathways and 224 down-regulated genes were assigned to 76 KEGG pathways (**Supplementary Table 8A**). In the top 20 enriched KEGG pathways, 11 pathways were significantly down-regulated and three pathways were significantly up-regulated under K^+^ deficiency (*P* < 0.05; Number of genes in one KEGG pathway ≥ 3) (**Figure 4**; **Supplementary Table 8A**). K^+^ stress led to significant up- or down-regulation of genes related to “mitogen-activated protein kinase (MAPK) signaling pathway-plant,” “plant hormone signal transduction,” and “starch and sucrose metabolism”. Pathways related to “plant-pathogen interaction,” “glycerophospholipid metabolism,” “alpha-linolenic acid metabolism,” “endocytosis,” “amino sugar and nucleotide sugar metabolism,” “glycerolipid metabolism,” “phosphatidylinositol signaling system,” “inositol phosphate metabolism,” and “valine, leucine and isoleucine degradation” pathways were down-regulated, whereas genes related to “plant circadian rhythm,” “phenylpropanoid biosynthesis,” “phagosome,” and “protein processing in endoplasmic reticulum” were up-regulated to resist K^+^ stress (**Figures 4** , **5**).

**Figure 6:**
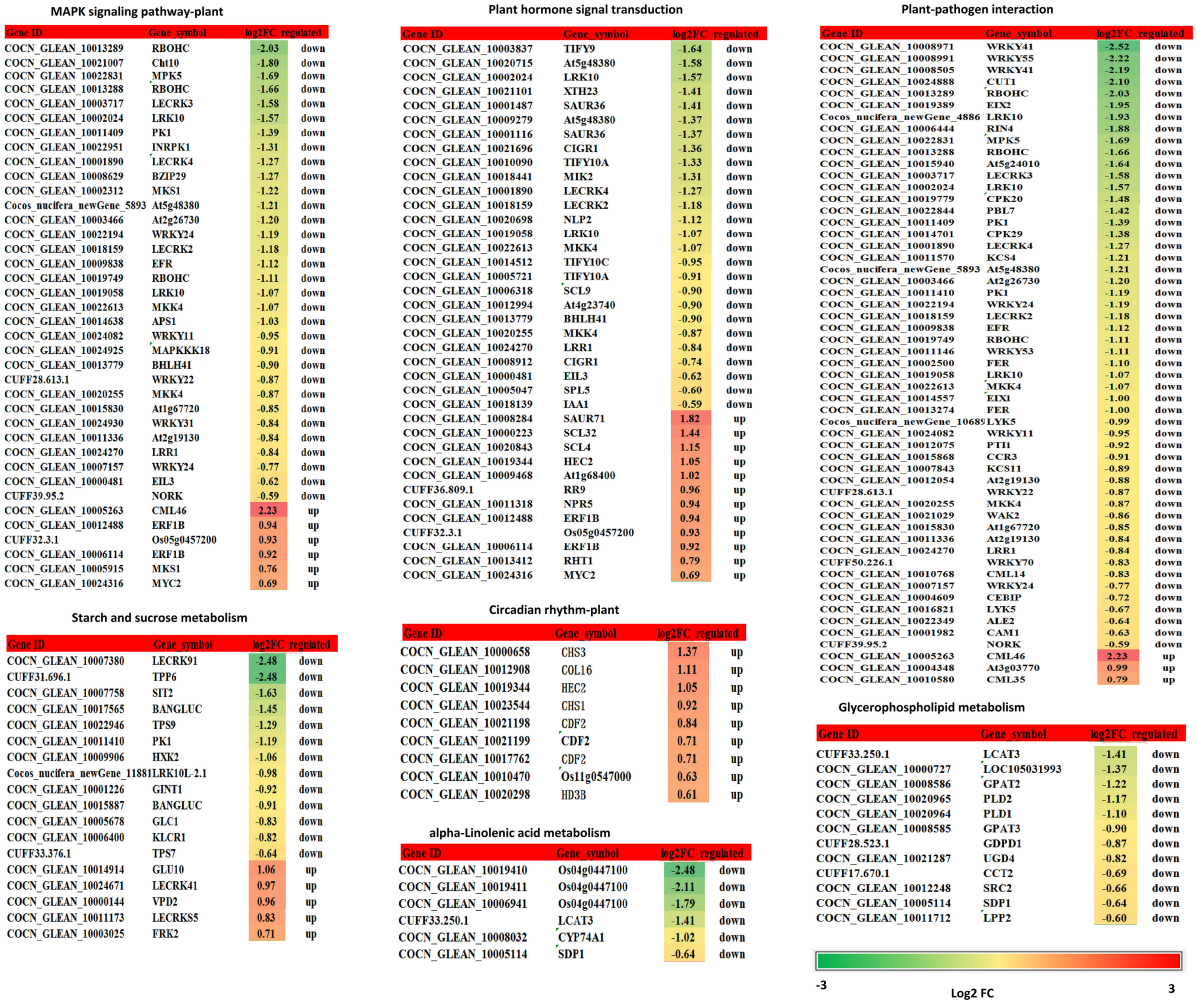
DEGs related to the MAPK signaling pathway - plant, starch and sucrose metabolism, plant hormone signal transduction, plant-pathogen interaction, glycerophospholipid metabolism, alpha-linolenic acid metabolism, circadian rhythm - plant. Changes in relative expression under K^+^ deficiency (log2FC based on mean RPKM in K_0_ vs. K_ck_) are shown as a color gradient from low (green) to high (red). Log2(FC) > 0 indicates upregulation, log2(FC) < 0 indicates downregulation, and log2(FC) = 0 indicates unchanged expression.

K^+^ deficiency stress may affect amino acid and sugar metabolism in coconut seedlings. According to DEG enrichment in the top 20 KEGG pathways, 38 DEGs were related to the MAPK signaling pathway-plant; 95% of these were down-regulated, especially, four genes (RBOHC (id:COCN_GLEAN_10013289, COCN_GLEAN_10013288), Cht10, MPK5, LECRK3) were down-regulated by –1.66 to 2.03 fold (log2FC) under K_0_ treatment (**Figure 6**). In contrast, probable calcium-binding protein (CML46) (log2FC = 2.23) was significantly up-regulated. Eighteen DEGs were related to starch and sucrose metabolism, 72% of which were down-regulated, including LECRK91 (log2FC = –2.48), TPP6 (log2FC = –2.48), and SIT2 (log2FC = –1.63) (**Figure 6**). Thirty-eight DEGs were involved in plant hormone signal transduction, 52% of which were down-regulated and 48% were up-regulated, with TIFY9 (log2FC = –1.64), At5g48380 (log2FC = –1.58), LRK10 (id: COCN_GLEAN_10019058) (log2FC = –1.57) being significantly down-regulated, and SAUR71 (log2FC = 1.82) being significantly up-regulated. Fifty-five DEGs were related to plant-pathogen interaction; 95% of them were down-regulated, including WRKY41 (COCN_GLEAN_10008971, COCN_GLEAN_10008505) (log2FC = –2.25 to –2.19), WRKY55 (log2FC = –2.22), CUT1 (log2FC = –2.10), RBOHC (log2FC = –2.03), EIX2 (log2FC = –1.95), and LRK10 (COCN_GLEAN_10002024) (log2FC = –1.93). Twelve DEGs were related to glycerophospholipid metabolism, and LCAT3 (log2FC = –1.41) was the most significantly down-regulated DEG. Six DEGs were related to alpha-linolenic acid metabolism, all of which were down-regulated; the most significantly down-regulated DEG was Os04g0447100 (log2FC = –1.7 to –2.48). Nine DEGs were related to the circadian rhythm pathway-plant; the most significant DEG was CHS3 (log2FC = 1.37) (**Figure 6**; **Supplementary Table 8B**).

### RNA-Seq validation of the RNA-Seq results by RT-qPCR

Thirteen genes were randomly selected to verify the RNA-seq results. The genes down-regulated (*P* < 0.05) under K starvation according to RNA-seq included ethylene-responsive transcription factor (ERF026), putrescine hydroxycinnamoyltransferase 1 (PHT1), probable WRKY transcription factor 41 (WRKY41), respiratory burst oxidase homolog protein C (RBOHC), ABC transporter G family member 39 (ABCG39), wall-associated receptor kinase 2 (WAK2), and L-type lectin domain-containing receptor kinase IX.1 (LECRK91). The up-regulated genes included CBL-interacting serine/threonine-protein kinases 14 and 25 (CIPK14, CIPK25), probable calcium-binding protein (CML46), putative potassium transporter 8 (HAK8), auxin-responsive protein (SAUR71), and chlorophyll a-b binding protein 91R (CAB91R). RT-qPCR was used to analyze the relative expression of selected genes. The relative expression changes (log2FC) of the 13 genes in three biological repeats as determined by RT-qPCR correlated well with the RNA-seq results (R^2^ = 0.987) (**Supplementary Figure 4**). These results confirmed the accuracy of the RNA-seq analysis in this study.

### Metabolome response to K^+^ deficiency

**Figure 7:**
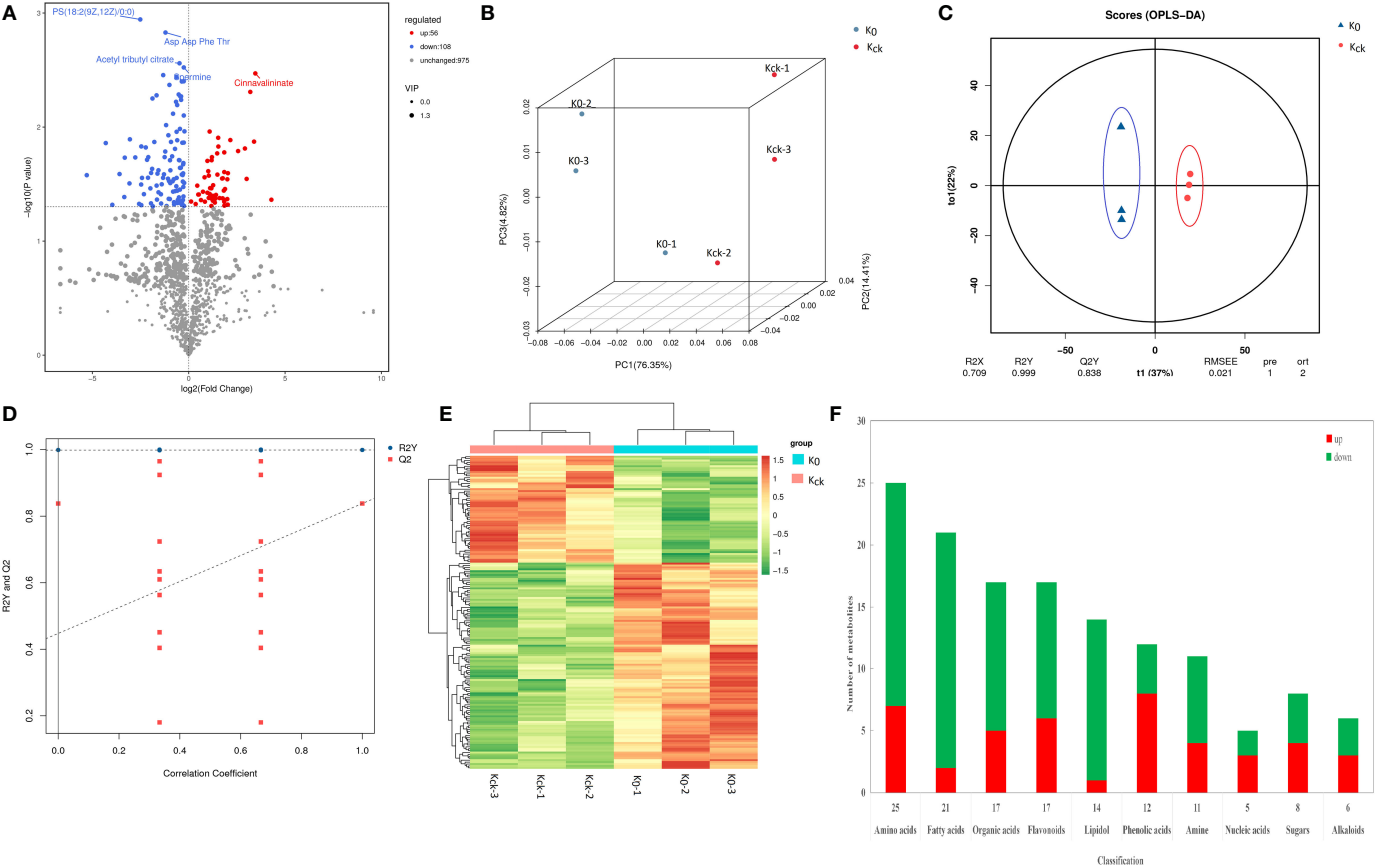
Analysis of DAMs in K_0_ vs. K_ck_. **(A)** Volcano plat of the DAMs. Each dot represents a metabolite. The X-axis represents the change in each substance (Log2), the Y-axis represents the *P*-value of the *t*-test (Log10), and the scatter size represents the variable importance of projection VIPvalue of the OPLS-DA model. down-regulated DAMs are indicated in blue, up-regulated DAMs are shown in red, and metabolites with insignificant differences are shown in grey. The first five qualitative metabolites were selected and labeled in the figure, based on their *P*-value. **(B)** Three-dimensional diagram of PCA. **(C)** OPLS-DA score model chart. R2X and R2Y represent the interpretation rate of the built model to the X and Y matrices, respectively, wherein the X matrix is the model input, i.e., the metabolite quantitative matrix, the Y matrix is the model output, i.e., the sample grouping matrix, and Q2 represents the prediction ability of the model, i.e., whether the built model can distinguish the correct sample grouping based on metabolite expression. The closer R2Y and Q2 are to 1 in the index, the more stable and reliable the model is. This model can be used to screen DAMs. Generally, Q2 > 0.5 indicates an effective model, and Q2 > 0.9 reflects an excellent model. **(D)** OPLS-DA model validation chart. The X-axis represents the similarity with the original model, the Y-axis represents the value of R2Y or Q2 (where R2Y and Q2 taken as 1 in the abscissa are the values of the original model), the blue and red points represent R2Y and Q2 of the model after Y replacement, respectively, and the dotted line is the fitted regression line. **(E)** Clustering heat map of the 164 DAMs. **(F)** Histogram of classification changes of different metabolites.

A total of 227 metabolites were detected in UPLC-MS/MS analysis of the two K^+^ treatment groups. There were 164 DAMs, including 108 down-regulated and 56 up-regulated DAMs, in the K_0_ vs. K_ck_ group (**Supplementary Table 9A**; **Figure 7A**). In PCA and OPLS-DA of the 164 DAMs, the groups were fairly well-separated (**Figures 7B–D**). A heatmap of the DAMs between the two groups showed a similar trend (**Figure 7E**). The repeatability among the three experimental (K_0_) and three control (K_ck_) sample groups was good, and the degree of separation between K_0_ and K_ck_ was significant. The DAMs were classified into seven categories: sugars (8), organic acids (17), amino acids (25), fatty acids (21), amines (12), fatty alcohols (14), nucleic acids (5), flavonoids (17), alkaloids (6), phenolic acids (12), and others (27) (**Figure 7F**; **Supplementary Table 9B**). Under K^+^ deficiency, 66% of the metabolites in coconut seedling leaves were down-regulated, which included 90% fatty acids, 93% fatty alcohols, 67% amines, 71% organic acids, 72% amino acids, 40% nucleic acids, 50% sugars, 65% flavonoids, 50% alkaloids, and 33% phenolic acids. In contrast, 34% of the metabolites were up-regulated, including 67% phenolic acids, 60% nucleic acids, 50% sugars, and 50% alkaloids. Notably, the levels of guanethidine, glycyl-threonine, isoleucyl-asparagine, 11.alpha.-hydroxyprogesterone, mevinolinic acid, tetrahydrodeoxycortisol, C16 sphingosine, and (+-)-lavandulol were 3–5 fold lower under K^+^ deficiency than under normal potassium fertilization. In contrast, the contents of procyanidin A1, S-nitroso-L-glutathione, PC(18:2(9Z,12Z)/16:0), cinnavalininate, and neohesperidin were 3–5 times higher under K^+^ deficiency than under normal potassium fertilization (**Supplementary Table 9B**). Among the 164 DAMs detected, the top 10 up-regulated DAMs were neohesperidin, cinnavalinine, PC(18:2(9Z,12Z)/16:0), S-Nitroso-L-glutathione, procyanidin A1, mevalonic acid, dihydro-5-methyl-2(3H)-thiophenone, 3-Hydroxycoumarin, 2,3’,4,6-tetrahydroxybenzophenone and acetamiprid. The top 10 down-regulated DAMs were (+-)-lavandulol, C16 Sphingosine, tetrahydrodeoxycortisol, mevinolinic acid, 11.alpha.-hydroxyprogesterone, isoleucyl-asparagine, glycyl-threonine, guanethidine, glycine Gln and 4-mercapto-4-methyl-2-pentanone (**Supplementary Table 9B**, **Supplementary Figure 5**)

**Figure 8:**
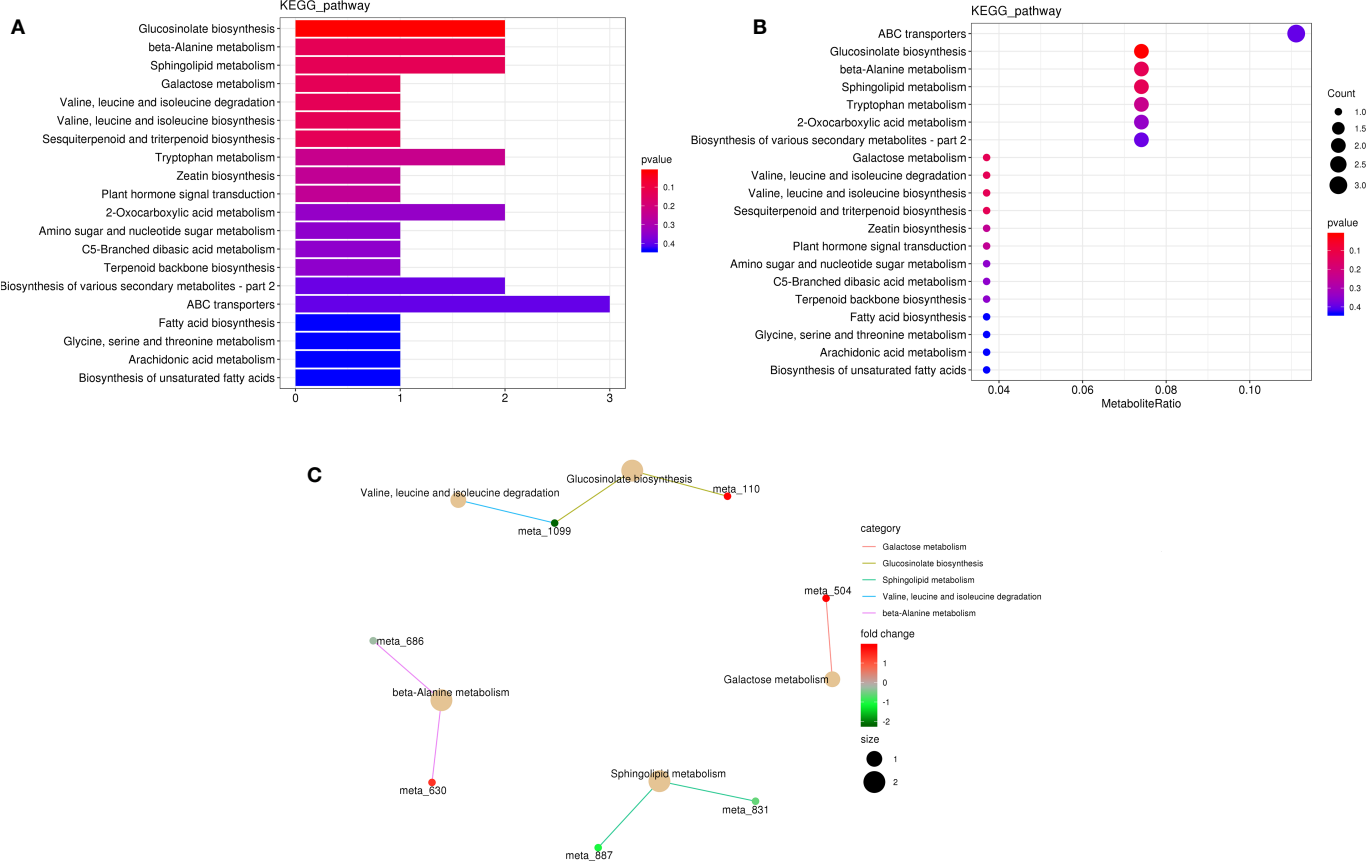
Metabolite pathway analysis in K_0_ vs. K_ck_. **(A)** Classification diagram of different metabolite pathways in each group. The X-axis indicates the number of DAMs annotated to a pathway, and the Y-axis indicates the name of the pathway. **(B)** Enrichment map of DAMs in KEGG pathways. The X-axis represents the ratio of the DAMs in a pathway to all DAMs with pathway annotation. **(C)** KEGG enrichment network diagram of DAMs. The light-yellow node indicates the pathway, and the small node connected to it indicates the specific metabolite annotated to the pathway. Color depth represents Log2(FC). The figure shows up to five pathways.

In KEGG pathway analysis, 227 metabolites (28 DAMs) were annotated to 40 KEGG pathways, and the DAMs (l-isoleucine, 2-oxo-5-methylthiopentanoic acid, l-anserine, spermine, phytosphingosine, 3-ketosphinganine, raffinose, capsidiol, cinnavalininate, and adenine; **Supplementary Table 10**) were mainly enriched in glucosinolate biosynthesis; beta-alanine metabolism; sphingolipid metabolism; galactose metabolism; valine, leucine, and isoleucine degradation; valine, leucine, and isoleucine biosynthesis; sesquiterpenoid and triterpenoid biosynthesis; tryptophan metabolism; zeatin biosynthesis; and plant hormone signal transduction pathway (**Figure 8**).

### Integrated metabolome and transcriptome analysis revealed crucial pathways involved in the response to K+ deficiency

**Figure 9:**
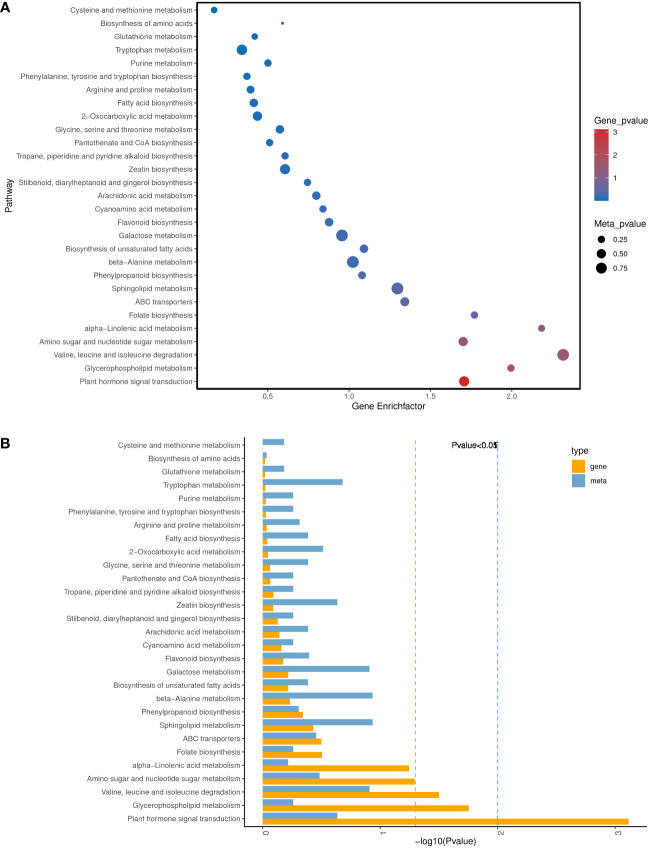
Analysis of KEGG pathways significantly enriched in DEGs/DAMs in K_0_ vs. K_ck_. **(A)** Bubble diagram of the top 30 KEGG pathways significantly enriched in DEGs/DAMs. The X-axis represents the Gene Enrich factor, the diff/background of genes in this pathway, and the Y-axis indicates the enrichment pathway. Dot size represents the significance of enrichment of the annotated DAMs in the pathway; the larger the dot, the more enriched it is. Dot color represents the significance of enrichment of DEGs in this pathway; the deeper the color, the more enriched they are. **(B)** Histogram of the top 30 KEGG pathways with significant enrichment of DEGs/DAMs. Each column represents a KEGG pathway. Yellow represents the transcriptome and blue represents the metabolome. Pathway names are indicated on the Y-axis, and the X- axis indicates the significance of enrichment of each pathway, that is, the FDR value taking the logarithm of the FDR.

**Figure 10:**
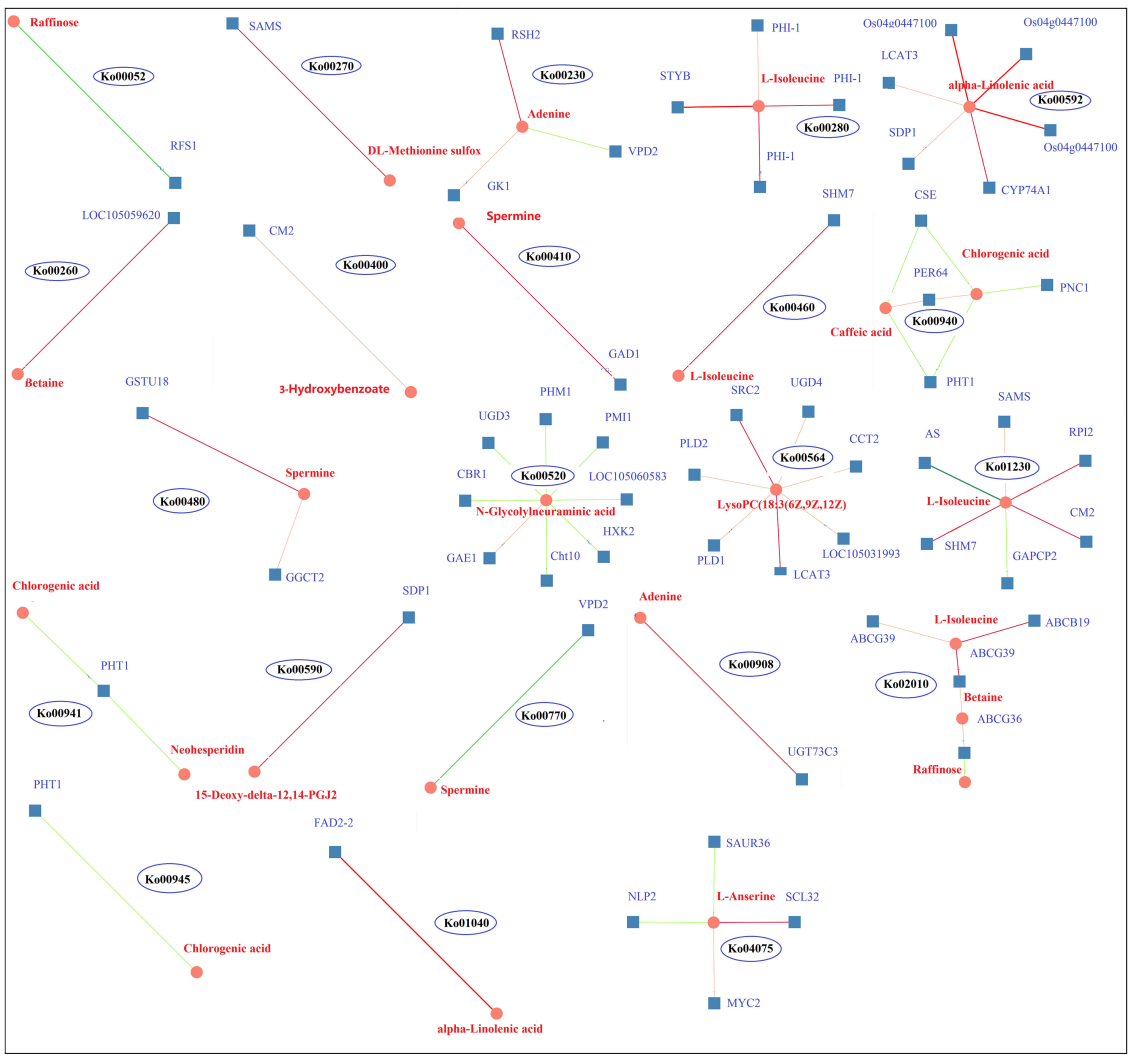
DEG and DAM correlation network diagram of 22 KEGG pathways in K_0_ vs. K_ck_. Circles represent metabolites, boxes represent genes, and values on the line represent correlation coefficients. Positive and negative correlation is indicated in red and green, respectively. The larger the correlation coefficient, the wider the line and the darker the color. The number in each ellipse indicates the ID of the KEGG pathway corresponding to the network diagram.

KEGG pathway enrichment analysis of the DEGs and DAMs showed that 28 pathways were enriched in K_0_/K_ck_ (**Supplementary Table 11**).Interestingly, among these pathways, those related to plant hormone signal transduction; glycerophospholipid metabolism; valine, leucine, and isoleucine degradation; amino sugar and nucleotide sugar metabolism; alpha-linolenic acid metabolism; folate biosynthesis; ABC transporters; sphingolipid metabolism; phenylpropanoid biosynthesis; and beta-alanine metabolism were significantly enriched under K^+^ deficiency (**Figure 9**). Based on the DAMs and DEGs, a screening was carried out according to the correlation coefficient (CC) and the relevant *P* value, with a screening threshold of | CC | > 0.70 and CC*P* < 0.05. Among nine quadrants, the patterns of DEGs and DAMs were consistent in quadrants 1, 3, 7, and 9 (**Supplementary Figure 6A**), and the regulation of genes and metabolites was positively or negatively correlated. Correlation analysis, hierarchical clustering, and correlation coefficient matrix heatmaps of the DAMs and DEGs showed a similar trend (**Supplementarys Figure 6B,C**). There were 1003 DEGs between the two groups, corresponding to 164 differential metabolites. We detected a significant correlation between 995 genes and 164 metabolites (*P* < 0.01, R^2^ > 0.8), identified 22 common KEGG pathways (metabolite and gene correlations), and drew a network diagram for correlation analysis of the metabolites and genes (**Figure 10**; **Supplementary Table 11**).

RFS1 and raffinose were enriched in galactose metabolism (Ko00052) and were negatively correlated; RFS1 was up-regulated and raffinose was down-regulated, suggesting that RFS1 may inhibit galactose metabolism. SAMS and DL-methionine sulfur co-enriched in the cysteine and methionine metabolism (Ko00270) pathway and were down-regulated and positively correlated. LOC105059620 and betaine were enriched in the glycine, serine, and threonine metabolism (Ko00260) pathway; both were down-regulated and positively correlated. STYB, PHI-1, and L-Isoleucine co-enriched in the valine, leucine, and isoleucine degradation (Ko00280) pathway, and the genes as well as metabolites were down-regulated, showing a positive correlation. SHM7 and L-Isoleucine co-enriched in the cyanoamino acid metabolism (Ko00460) pathway, and SHM7 was down-regulated and positively correlated with L-Isoleucine. CM2, HM7, RPI2, SAMS, AS, GAPCP2, and L-Isoleucine enriched in amino acid biosynthesis (Ko01230) pathway; CM2, SHM7, RPI2, and SAMS were down-regulated and positively correlated with L-Isoleucine, whereas AS and GAPCP2 were up-regulated and negatively correlated with L-Isoleucine. CM2 and 3-hydroxybenzoate were enriched in the phenylalanine, tyrosine, and tryptophan biosynthesis (Ko00400) pathway, both were down-regulated and positively correlated. ABCG39, ABCB19, ABCG36, L-Isoleucine, betaine, and raffinose were enriched in the ABC transporters (Ko02010) pathway; ABCG39, ABCB19, ABCG36, L-Isoleucine, and betaine were down-regulated; ABCG39 and ABCB19 were positively correlated with L-Isoleucine; ABCG39 and ABCG36 were positively correlated with betaine; and ABCG36 was negatively correlated with raffinose. UGD3, CBR1, PMI1, PHM1, LOC105060583, HXK2, Cht10, GAE1, and N-Glycdyneuramicacid were enriched in the amino sugar and nuclear sugar metabolism (Ko00520) pathway; UGD3, CBR1, PMI1, PHM1, LOC105060583, HXK2, and Cht10 were down-regulated and negatively correlated with N-Glycdyneuramic acid, whereas GAE1 was up-regulated and positively correlated with the genes regulating the synthesis and metabolism of amino acid analogs. Os04g0447100, LCAT3, SDP1, and alpha-Linolenic acid were enriched in the alpha-Linolenic acid metabolism (Ko00592) pathway, all of which were down-regulated and positively correlated, and thus may regulate alpha-Linolenic acid metabolism. FAD2-2 and alpha-Linolenic acid were enriched in the biosynthesis of unsaturated fatty acids (Ko01040) pathway, and both were down-regulated and positively correlated. SDP1 and 15-Deoxy-delta-12,14-PGJ2 were enriched in the arachidonic acid metabolism (Ko00590) pathway; both were down-regulated and positively correlated. SRC2, LCAT3, UGD4, CCT2, LOC105031993, PLD1, PLD2, and LysoPC(18:3(6Z,9Z,12Z)) were enriched in the glycerophospholipid metabolism (Ko00564) pathway; the genes were down-regulated and positively correlated with LysoPC(18:3(6Z,9Z,12Z)), indicating that these genes regulate the synthesis and metabolism of fatty acid analogs. RSH2, GK1, VPD2, and adenine were co-enriched in the purine metabolism (Ko00230) pathway; RSH2, GK1, and adenine were down-regulated and positively correlated, whereas VPD2 was up-regulated and negatively correlated with adenine. RSH2, GK1, and VPD2 can jointly regulate purine metabolism. UGT73C3 and adenine were enriched in the zeatin biosynthesis (Ko00908) pathway, and both were down-regulated and positively correlated. GAD1 and spermine were enriched in the beta-Alanine metabolism pathway (Ko00410); both were down-regulated and positively correlated. GSTU18, GGCT2, and spermine were enriched in the glutathione metabolism pathway (Ko00480) and showed a downward trend; GSTU18 and GGCT2 positively correlated with spermine levels. VPD2 and spermine were enriched in the pantothenate and CoA biosynthesis (Ko00770) pathway and showed a negative correlation. SCL32, MYC2, SAUR36, NLP2, and L-Anserine were enriched in the plant hormone signal transformation (Ko04075) pathway; SCL32, MYC2, and L-Anserine were up-regulated and positively correlated, whereas SAUR36 and NLP2 were down-regulated and negatively correlated with l-anserine; therefore, these genes may regulate glutathione metabolism; purine, zeatin, pantothenate, and CoA biosynthesis; and plant hormone signal transformation. CSE, PHT1, PNC1, PER64, caffeic acid, and chlorogenic acid were enriched in the phenylpropanoid biosynthesis (Ko00940) pathway; CSE, PHT1, and PNC1 were down-regulated, while PER64, caffeic acid, and chlorogenic acid were up-regulated. CSE and PHT1 were negatively correlated with caffeic acid and chlorogenic acid, and PNC1 was negatively correlated with chlorogenic acid; however, PER64 was positively correlated with caffeic acid and chlorogenic acid. PHT1, chlorogenic acid, and neohesperidin were enriched in the flavonoid biosynthesis (Ko00941) pathway, and PHT1 was negatively correlated with chlorogenic acid and neohesperidin. PHT1 and chlorogenic acid were negatively correlated in stilbenoid, diarylheptanoid, and ginger biosynthesis (Ko00945). The results showed that the genes encoding CSE, PHT1, PNC1 and PER64 regulated phyloproponoid, flavonoid, stilbenoid, diarylheptanoid, and ginger biosynthesis.

**Figure 11:**
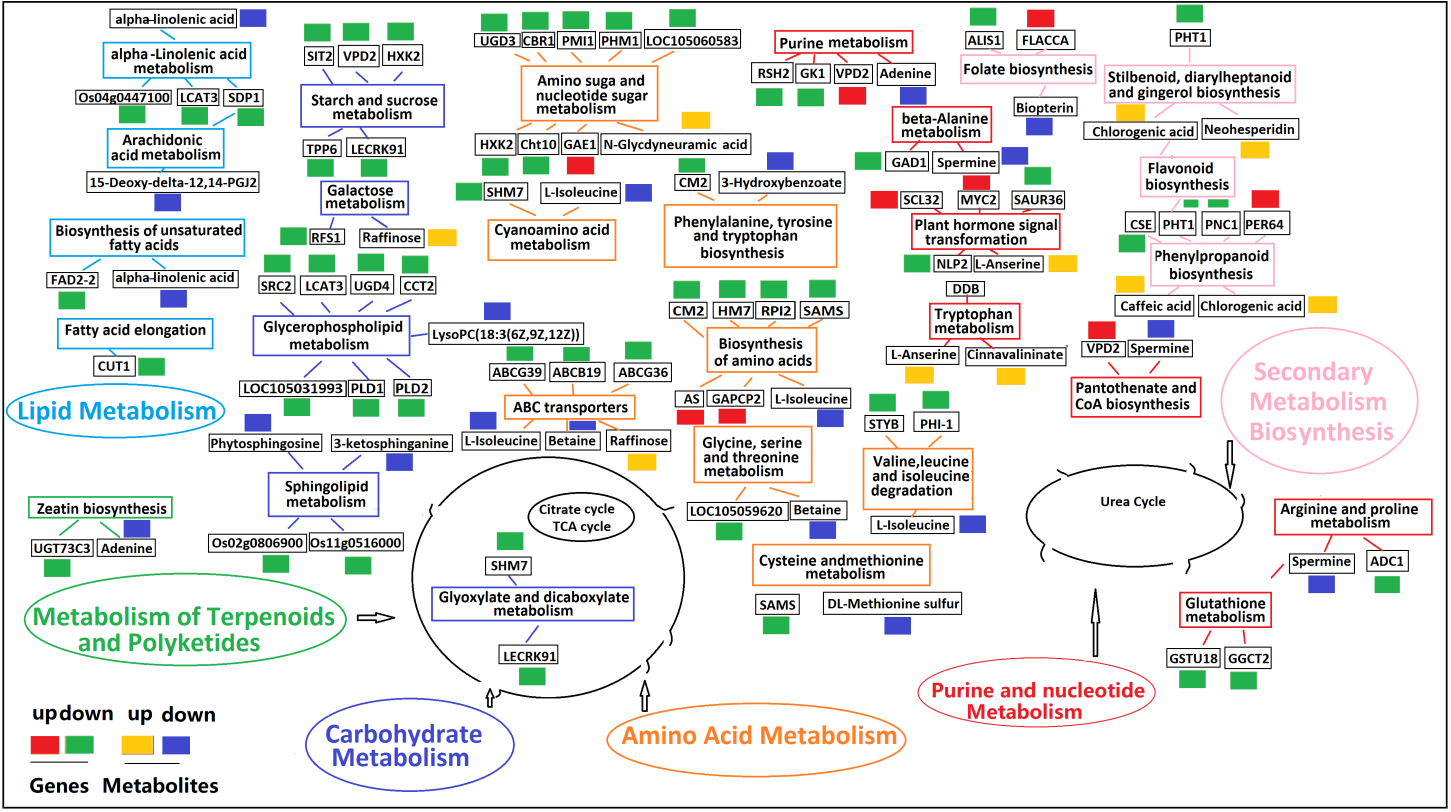
Profiles of DEGs and DAMs in flavonoid biosynthetic pathways in K_0_ vs. K_ck_. The boxes in the pathway represent DEGs or DAMs. Red and green represent up- and down-regulated genes, respectively. Yellow and blue represent up- and down-regulated metabolites, respectively. Boxes of the same color represent the relevant pathway.

A metabolic network diagram based on 21 DAMs and 133 DEGs under K^+^ deficiency stress is shown in **Figure 11**. The network clearly shows that the down-regulated DAMs (62%) and DEGs (75%) were mainly involved in the metabolism of fatty acids, lipids, amino acids, organic acids, amines, and flavonoids, which was in line with the finding that the CF and SF contents were significantly reduced by 56.82% and 24.83%, respectively. This may be due to a significant reduction in several intermediates of the tricarboxylic acid cycle under K^+^ deficiency. The down-regulated metabolites included fat acids related to 15-Deoxy-delta-12,14-PGJ2, LysoPC(18:3(6Z,9Z,12Z)), cis-9-Palmitoleic acid, 3-Hydroxybenzoate, lipidol-related 3-ketosphingonine, phytosphingosine, amino acid-related L-Isoleucine, beta, DL-Methionine sulfur, amine-related spermine, adenine, organic acid-related alpha-Linolenic acid, and flavonoid-related bioprotein. Levels of sugar- and phenolic acid-related metabolites were mostly increased, especially those involved in the pentose phosphate pathway (PPP), such as raffinose, chlorogenic acid, and caffeic acid, which may be due to glycolysis and the synthesis and metabolism of secondary products under K_0_ conditions to adapt to K stress. Twenty-two KEGG pathways were enriched, including plant hormone signal transport, glycerophospholipid metabolism; valine, leucine, and isoleucine degradation; amino sugar and nuclear sugar metabolism; alpha-Linolenic acid metabolism; folate biosynthesis; ABC transporters; sphingolipid metabolism; phenylpropanoid biosynthesis; and beta-Alanine metabolism. in the metabolic pathways, there was a significant positive correlation between DEGs and DAMs, with most DEGs and DAMs being down-regulated.

## Discussion

Mineral nutrients (N, P, and K) are crucial for plant growth and development, and their effects on plant growth, morphology, physiology, transcriptome, and metabolome under different exogenous nutrient conditions have been reported (Pettigrew, 2008; Hafsi et al., 2014; Mo et al., 2019; Wang et al., 2019; Ma et al., 2020; Ding et al., 2021). In our study, the biomass of coconut seedlings significantly decreased under K^+^ deficiency. We also analyzed the transcriptomic and metabolic changes under long-term K^+^ deficiency. Most DEGs were related to pathways such as signal regulation and transport, primary and secondary metabolism, and plant-pathogen interactions. DAMs induced by K^+^ deficiency were mainly involved in primary and secondary metabolism, including the metabolism of fat acids, amino acids, sugars, lipidols, organic acids, amines, flavonoids, phenolic acids, and alkaloids. The results showed that genes related to MAPK signaling, plant hormone signal transduction, starch and sucrose metabolism, plant-pathogen interaction, glycerophospholipid metabolism, and plant circadian rhythm may play an important role in the response of coconut seedling leaves to K^+^ deficiency (**Supplementary Tables 6, 8, 9**).

### Developmental responses to K^+^ deficiency

K^+^ deficiency limited the growth of coconut seedlings and led to significant reductions in whole-plant biomass and fruit yield. The application of K^+^ fertilizer promotes the growth of coconut trees and increases total biomass and yield, indicating that K^+^ directly affects the biomass and yield of coconut trees (Mohandas, 2012a). Most studies have focused on the effects of K^+^ on coconut growth and yield, rather than on the growth, physiology, and metabolic regulation (Chen, 2005). Due to the lack of K^+^, the height of coconut seedlings decreased, which significantly limited plant growth and reduced dry weight (**Supplementary Table 2**). This indicates that low-K^+^ stress significantly reduced seedling biomass. In green plants, photosynthesis is an important process that provides the necessary energy sources for metabolism (Chen et al, 2015; Zhang et al., 2020). Low-K^+^ stress inhibits the photosynthetic activity of plants, leading to slower metabolism, thus affecting plant growth and development (Kanai et al., 2011; Amanullah. et al., 2016). According KEGG pathway annotation, numerous significantly down-regulated DEGs were involved in plant MAPK signaling, starch and sucrose metabolism, phenylpropanoid biosynthesis, ABC transporters, and photosynthesis (**Figures 4**, **5**; **Supplementary Table 8**). Therefore, low-K^+^ stress inhibits the photosynthetic activity, signal transduction and regulation, primary product synthesis, and metabolism of coconut seedlings, thus affecting their growth and development. K^+^ plays an important role in photosynthesis and ATP generation and severe K^+^ deficiency suppresses photosynthesis (Kanai et al., 2011; Huang et al., 2013). Roots have low K^+^ absorption efficiency, which significantly reduces K content in leaves (48.36% lower than in the control), inhibits photosynthesis, and suppresses plant growth (**Supplementary Table 3**; Battie-Laclau et al., 2014; Ma et al., 2020). Leaves and stems act as sinks for K^+^ and carbon assimilation during plant growth, and plant growth retardation acts as a feedback signal to K^+^ deficiency (Kanai et al., 2007). Similar K^+^ deficiency responses have been observed in tomato, sugarcane, and *Eucalyptus grandis* (Hartt, 1969; Kanai et al., 2007; Ployet et al., 2019). Under K^+^ deficiency, the SPAD value was significantly lower than that in control seedlings, and K^+^ deficiency reduced plant growth and biomass, which may be due to an increase in reactive oxygen species in coconut seedlings caused by K^+^ deficiency, leading to a reduction in chlorophyll. The expression of a chlorophyll-related gene (encoding ferredoxin nitrate reductase) was down-regulated in the leaves of coconut seedlings (**Figure 1**; **Supplementary Table 6**), which may have contributed to the decrease in the SPAD value of K_0_ seedlings.

The activity of antioxidant enzymes in plant cells is stimulated by environmental stress, and reactive oxygen-scavenging enzymes, such as SOD, CAT, and POD, play an important role in plant antioxidant defense (Rahimizadeh et al., 2007). Under K^+^ deficiency, the activities of CAT, POD, and SOD in the leaves of coconut seedlings were significantly decreased (**Figure 2**); this may be due to the decreased expression of genes (IBS1, NSL1, PXC3, ORM1, LOC105044768, Os02g0806900, Os11g0516000) related to the synthesis and metabolism of proteins or proteases under K^+^ deficiency stress (**Supplementary Tables 6, 8**). Furthermore, the Pro content was significantly reduced, and genes and metabolites related to purine metabolism were down-regulated, suggesting that the downregulation of Pro metabolism-related genes and metabolites under K^+^ deficiency significantly reduced the Pro content in coconut leaves. Low-K^+^ stress also inhibited MDA activity, which may have led to MDA accumulation under K^+^ deficiency (**Figure 2**; **Supplementary Tables 6, 8**). Studies have shown that low K levels can induce lateral root growth in maize *via* regulating genes involved in nutrient utilization, hormones, and transcription factors (Zhao et al., 2016; Ma et al., 2020). In this study, except for ABA, endogenous hormone levels were significantly decreased under K^+^ deficiency stress, which is consistent with the finding that most genes involved in plant hormone metabolism were significantly down-regulated, whereas some genes related to aging and abscission were significantly up-regulated (**Figure 2**; **Supplementary Tables 6, 8**), indicating that K^+^ deficiency stress has a strong impact on endogenous hormones in coconut seedling leaves.

### Transcription responses to K^+^ deficiency

Studies have examined the transcriptome spectrum of plant responses to K^+^ deficiency (Ruan et al., 2015; Zhao et al., 2018; Ployet et al., 2019; Ma et al., 2020; Yang et al., 2021). We analyzed the transcriptional changes in coconut seedling leaves under K^+^ deficiency and K^+^ sufficiency, and 1003 DEGs were identified between the two groups. GO enrichment analysis showed that the largest proportion of DEG enrichment was found in the MF category. In the BP category, most of the DEGs down-regulated under K^+^ deficiency were related to “sesquiterpene biosynthetic process,” “cellular sphingolipid homeostasis,” “negative regulation of ceramide biosynthetic process,” “sphingosine biosynthetic process,” “regulation of jasmonic acid mediated signaling pathway,” and “ceramide metabolic process”. In terms of CC, the down-regulated DEGs were mainly related to “integral component of membrane,” “serine C-palmitoyltransferase complex,” “SPOTS complex,” and “plasma membrane”. In the MF category, the down-regulated DEGs were mostly involved in “cyclase activity,” “protein kinase activity,” “serine C-palmitoyltransferase activity,” “ATP binding,” “magnesium-dependent protein serine/threonine phosphatase activity,” “protein serine/threonine kinase activity,” “protein serine/threonine phosphatase activity,” “calcium ion binding,” “sphingosine-1-phosphate phosphatase activity,” “transferase activity, transferring glycosyl groups,” and “polysaccharide binding,” whereas the up-regulated DEGs were mainly related to “nucleus,” “transcription factor activity, sequence-specific DNA binding,” “sequence-specific DNA binding,” and “DNA binding” (**Figure 3**). In these enriched GO terms, genes involved in plant hormone signal transduction (TIFY9), plant-pathogen interaction (WRKY41, WRKY55, ERF026), signal transduction mechanisms (WAK2, PLL5), spliceosome and endocytosis (LRK10, Hsp70-4), ABC transporters (ABCG39), starch and sucrose metabolism (LECRK91), MAPK signaling pathway-plant (RBOHC), fatty acid elongation (CUT1), amino acid transport and metabolism (URGT2, GALT6), plant-pathogen interaction (EIX2), and protein kinase/protein domain (IBS1, NSL1, PXC3, ORM1, LOC105044768, Os02g0806900, Os11g0516000) were significantly down-regulated. However, the gene (CML46) encoding a probable calcium-binding protein related to MAPK signaling was significantly up-regulated (**Supplementary Table 7**). Transcriptome studies on the effects of nutrient stress on crops have shown that DEGs in these processes may play an important role in plant adaptation to P deficiency (Li et al., 2009). In contrast to our findings in coconut, in wheat subjected to low-P stress, DEGs related to amino acid metabolism, photosynthesis, carbohydrate metabolism, and organic acid metabolism were highly up-regulated (Wang J. et al., 2019). The expression of genes related to organic and amino acid metabolism was significantly altered in oats under P deficiency (Wang et al., 2018). In maize seedlings under K^+^ deficiency stress, MAPK signaling pathway-plant, signal transduction mechanisms, plant hormone signal transduction, and amino acid transport and metabolism were the mainy enriched pathways, and related gene expression was significantly altered (Xiong et al., 2022) In our study, in coconut seedling leaves under K^+^ deficiency stress, “MAPK signaling pathway-plant”, “signal transduction mechanisms”, “plant hormone signal transduction”, “amino acid transport and metabolism”, “plant-pathogen interaction”, “starch and sucrose metabolism”, “spliceosome”, “endocytosis”, “ABC transporters”, “fatty acid elongation”, and “protein kinase/protein domain” pathways were affected. In particular, genes related to “MAPK signaling pathway-plant”, “signal transduction mechanisms”, “plant hormone signal transduction”, “amino acid transport and metabolism” pathways were significantly down-regulated (**Supplementary Tables 7, 8**). Genes involved in plant hormone signal transduction affect the content and distribution of plant hormones, thus regulating plant growth (Ma et al., 2020). Therefore, we confirmed that MAPK signaling, plant hormone signal transduction, and transcription factors may be key regulators of the response of coconut seedlings to K^+^ deficiency.

KEGG pathway enrichment analysis showed that K^+^ deficiency stress affected genes were involved in “MAPK signaling pathway-plant”, “plant hormone signal transduction”, “starch and sucrose metabolism”, “plant-pathogen interaction,” “glycerophospholipid metabolism”, “alpha-Linolenic acid metabolism”, “endocytosis”, “amino sugar and nucleotide sugar metabolism”, “glycerolipid metabolism”, “phosphatidylinositol signaling system”, “inositol phosphate metabolism”, “valine, leucine and isoleucine degradation”, “circadian rhythm-plant”, “phenylpropanoid biosynthesis”, “phagosome” and “protein processing in endoplasmic reticulum” (**Figures 4** **5**; **Supplementary Table 8**). Under K^+^ deficiency, genes related to “MAPK signaling pathway-plant”, “plant hormone signal transduction”, “starch and sucrose metabolism” pathways in coconut seedling leaves were significantly modulated. For example, in the MAPK signaling pathway-plant, RBOHC, Cht10, MPK5, and LECRK3 were significantly down-regulated, whereas CML46, encoding a probable calcium-binding protein, was significantly up-regulated. The significantly down-regulated DEGs in plant hormone signal transduction were TIFY9, At5g48380, and LRK10, whereas SAUR71 was significantly up-regulated. The significantly down-regulated DEGs in starch and sucrose metabolism were LECRK91, TPP6, and SIT2. In addition, genes involved in plant-pathogen interactions (WRKY41, WRKY55, RBOHC, CUT1, EIX2), alpha-linolenic acid metabolism (putative lipoxygenase, Os04g0447100), endocytosis (HSP70-4, LRK10), amino sugar and nucleotide sugar metabolism (Cht10), glycerolipid metabolism (LOC105048065), and valine, leucine, and isoleucine degradation (PHI-1) were significantly down-regulated (**Supplementary Table 8**), indicating that these pathways were suppressed under K^+^ deficiency stress. However, genes in the circadian rhythm-plant (CHS3) and phenylpropanoid biosynthesis (CAD6) pathways (**Supplementary Table 8**) were up-regulated, which indicates that these pathways are enriched to resist K^+^ deficiency stress.

Under K^+^ deficiency, “MAPK signaling pathway-plant”, “plant hormone signal transduction”, “starch and sucrose metabolism”, “plant-pathogen interaction”, “glycerophospholipid metabolism” and “circadian rhythm-plant” were significantly enriched pathways (**Figure 5**; **Supplementary Table 8**). The significantly down-regulated genes involved in “MAPK signaling pathway-plant”, “plant hormone signal transduction”, “starch and sucrose metabolism”, “plant-pathogen interaction”, “glycerophospholipid metabolism” are mainly involved in the signal regulation process, transportation and primary metabolism, and the metabolism of disease resistant interacting substances (fat acids, lipidols, amino acids, organic acids, and amines, among others) (**Figure 11**; **Supplementary Table 10**). In addition, MAPK signaling is related to aging and apoptosis, which may explain why leaves have yellow or brown edges and tips under K^+^ deficiency (Sun et al., 2015). This study also showed that the CF content decreased by 56.82% and the SP content decreased by 24.83% under K^+^ deficiency (**Figure 1**). Genes related to circadian rhythm and metabolism of phenolic acids, flavonoids, and other substances were significantly up-regulated (**Figure 11**; **Supplementary Table 10**), which may be attributed to the fact that coconut seedlings can enhance some of these metabolites to resist K^+^ deficiency through the autoimmune effect. The genes involved in plant hormone signal transduction affect the content and distribution of plant hormones, thus regulating plant growth (Takahashi et al., 2005; Argyros et al., 2008; Ma et al., 2020). This study showed that relevant genes and metabolites in plant hormone signal transduction were significantly down-regulated, which is consistent with the significantly reduced contents of IAA, GA, and ZR in coconut seedling leaves (**Figure 2**; **Supplementary Table 8**). Transcription factors play indispensable roles in regulating the response to biotic and abiotic stresses (Ma et al., 2012; Zhang et al., 2017). In coconut seedling leaves under K^+^ deficiency, 71 transcription factors were differentially expressed, including WRKY, RLK, AP2/ERF, C2C2, Tify, bHLH, NAC, HB, GRAS, FAR1, MYB, bZIP, and PLATZ; among these, WRKY, RLK, Tify, and NAC were significantly down-regulated, and AP2/ERF, C2C2, MYB, and PLATZ were significantly up-regulated (**Supplementary Figure 7**).

### Metabolic responses to K^+^ deficiency

Metabonomic analysis showed that DAMs under K^+^ deficiency were mainly enriched in “glucosinolate biosynthesis,” “beta-Alanine metabolism,” “sphingolipid metabolism,” “galactose metabolism,” “valine, leucine and isoleucine degradation,” “valine, leucine and isoleucine biosynthesis,” “sesquiterpenoid and triterpenoid biosynthesis,” “tryptophan metabolism,” “zeatin biosynthesis” and “plant hormone signal transduction” pathways (**Figure 8**). DAMs (S-Nitroso-L-glutathione, glutaminyl-Asparagine, N-Glycolylneuraminic acid) related to amino acids were significantly up-regulated, whereas Isoleucyl-Asparagine, Glycyl-Threonine, Gly-Gln, L-Isoleucine, and Lysyl-Lysine were significantly down-regulated. Sugar-related raffinose was significantly up-regulated, whereas C-6 Ceramide was significantly down-regulated. Amine-related DAMs (cinnavalinine, L-anserine, procyanidin A1, and acetamiprid) were significantly up-regulated, whereas 13Z docosamide, anandamide, and adenine were significantly down-regulated. Purpurin, which is related to nuclear acids, was significantly up-regulated, whereas guanethidine was significantly down-regulated. DAMs (mevalonic acid, 2-Oxo-5-methylthiopentanoic acid, caffeic acid, 5-methyltetrahydrofolate) related to organic acids were significantly up-regulated, whereas alpha-lienolenic acid, mevinolinic acid, 9-hydroxy-10E, 12,15Z-Octadecatrienonic acid, 11(Z),14(Z),17(Z)-Ecosatrienoic acid were significantly down-regulated. A DAM (PC(18:2(9Z,12Z)/16:0) related to fatty acids was significantly up-regulated, whereas 13S HpOTrE (gamma), PS(18:2(9Z,12Z)/0:0), LysoPE(18:2(9Z,12Z)/0:0), and PC(18:2(9Z,12Z)/P-16:0) were significantly down-regulated. Nor-psi-tropine associated with lipidol was significantly up-regulated, whereas C16 Sphingosine, 1-Linoleoylglycophorophospholine, (+-)-lavandulol, 6-[5]-ladderane-1-hexanol, and tetrahydrooxygenycortisol were significantly down-regulated. DAMs (neohesperidin, dihydro-5-methyl-2 (3H)-thiophene, 2,3’, 4,6-tetrahydroxybenzophene) related to flavonoids were significantly up-regulated, whereas 11-alpha-hydroxyprogesterone and 4-mercapto-4-methyl-2-pentanone were significantly down-regulated. DAMs (6’’-O-acetylgenistein, chlorogenic acid, and benzaldehyde) associated with phenolic acids were significantly up-regulated, whereas acetal R and MG(0:0/18:3(9Z,12Z,15Z)/0:0) were significantly down-regulated. DAMs (3-hydroxycoumari and cytidine 5’-diphosphocholine) related to alkaloids were significantly up-regulated, whereas vanillin was significantly down-regulated (**Supplementary Table 9**). It can be concluded that K^+^ deficiency stress greatly affects the synthesis and expression of metabolites, including amino acids, sugars, amines, nucleic acids, fatty acids, flavonoids, phenolic acids, and alkaloids, in the leaves of coconut seedlings.

In addition, 66% of the metabolites were down-regulated, which included 90% fatty acids, 93% lipids, 67% amines, 71% organic acids, 72% amino acids, 40% nucleic acids, 50% sugars, 65% flavonoids, 50% alkaloids, and 33% phenolic acids, whereas 34% of the metabolites were up-regulated, including 67% phenolic acids, 60% nucleic acids, 50% sugars, and 50% alkaloids (**Figure 7**, **Supplementary Table 9**). In different substances, whether up-or down-regulated, each metabolite, whether up- or down-regulated, plays a different role and plays a very important role in response to low-K^+^ stress. For example, sugars such as raffinose, radish sugar, and glucosamine are accumulated in low-K^+^ stress, and are also detected in plants under P or cold stress (Cook et al., 2004; Ding et al., 2021; Xiong et al., 2022). Flavonoids and phenols are major secondary metabolites involved in plant immunity (Wang and Wu, 2013). Under K^+^ deficiency, flavonoid and phenolic levels changed significantly. RT-qPCR analysis showed that the expression of genes involved in the biosynthesis of flavonoids and phenolic substances (**Supplementary Figure 4**), which can activate enzymes and play an important role in protein synthesis (Hafsi et al., 2017), changed accordingly. Under K^+^ deficiency, the contents of CF and SP in coconut seedling leaves were decreased, and metabonomic analysis also showed that 90% of fatty acids and 72% of amino acids were down-regulated (**Figure 1**; **Supplementary Table 9**). This is inconsistent with the findings of previous studies that reported increased amino acid accumulation under K^+^ and P deficiency (Wang et al., 2012, Hernandez et al., 2007; Pant et al., 2015; Mo et al., 2019; Ding et al., 2021; Xiong et al., 2022). This may be because the lack of K^+^ in coconut seedlings inhibits carbon metabolism and affects amino acid accumulation. In addition, amino acid transport-related genes are reportedly down-regulated in response to K^+^ deficiency, which is accompanied by suppressed activity of transmembrane transport proteins and ATPases (**Supplementary Table 6**).

### Comparative transcriptome and metabolome responses to K^+^ deficiency

The comparative analysis of transcriptional and metabolic responses to K^+^ deficiency showed that DEGs and DAMs were mainly enriched in “plant hormone signal transduction,” “glycerophospholipid metabolism,” “valine, leucine and isoleucine degradation,” “amino sugar and nucleotide sugar metabolism,” “alpha-Linolenic acid metabolism,” “folate biosynthesis,” “ABC transporters,” “sphingolipid metabolism,” “phenylpropanoid biosynthesis” and “beta-alanine metabolism” pathways. In addition, the DEGs and DAMs were largely significantly correlated. For example, STYB, PHI-1, and L-isoleucine were co-enriched in the valine, leucine, and isoleucine degradation (Ko00280) pathway, and both, the genes and metabolites were down-regulated, showing a positive correlation. ABCG39, ABCB19, ABCG36, L-isoleucine, betaine, and raffinose were enriched in the ABC transporter (Ko02010) pathway; ABCG39, ABCB19, ABCG36 L-isoleucine, and betaine were down-regulated; ABCG39 and ABCB19 were positively correlated with L-isoleucine; ABCG39 and ABCG36 were positively correlated with betaine; and ABCG36 was negatively correlated with raffinose. UGD3, CBR1, PMI1, PHM1, LOC105060583, HXK2, Cht10, GAE1, and N-Glycdylneuraminic acid were enriched in the amino sugar and nuclear sugar metabolism (Ko00,520) pathway. UGD3, CBR1, PMI1, PHM1, LOC105060583, HXK2, and Cht10 were down-regulated and negatively correlated with N-Glycdylneuraminicacid, whereas GAE1 was up-regulated and positively correlated with N-Glycdylneuraminicacid (**Figure 10**; **Supplementary Table 11**). The synthesis and metabolism of amino acid analogs and ABC transporters have been discussed above (**Supplementary Table 11**). Amino acids and ABC transporters play important roles in K^+^ uptake and transport under K^+^ deficiency (Xie et al., 2020) and regulate the cellular K^+^ content to promote permeability (Cuin and Shabala, 2007). This study showed that genes and metabolites related to amino acids and ABC transporters were significantly modulated under K^+^ deficiency stress (**Supplementary Tables 8, 9, 11**), which indicates that K^+^ absorption and transport mechanisms are greatly affected by K^+^ deficiency. Os04g0447100, LCAT3, SDP1, and alpha-Linolenic acid were enriched in the alpha-Linolenic acid metabolism (Ko00592) pathway, and all were down-regulated. Os04g0447100, LCAT3, and SDP1 were positively correlated with alpha-Linolenic acid. Therefore, these genes may regulate alpha-Linolenic acid metabolism. SRC2, LCAT3, UGD4, CCT2, LOC105031993, PLD1, PLD2, and LysoPC(18:3(6Z,9Z,12Z)) were enriched in the glycerophospholipid metabolism (Ko00564) pathway, and the relevant genes and metabolites were down-regulated; however, SRC2, LCAT3, UGD4, CCT2, LOC105031993, PLD1, and PLD2 were positively correlated with LysoPC(18:3(6Z,9Z,12Z)), indicating that these genes and metabolites may regulate the synthesis and metabolism of fatty acid analogs. RSH2, GK1, VPD2, and adenine were co-enriched in the purine metabolism (Ko00230) pathway. RSH2, GK1, and adenine were down-regulated, and RSH2 and GK1 were positively correlated with adenine, whereas VPD2 was up-regulated and negatively correlated with adenine (**Figure 10**; **Supplementary Table S11**). Putrescine is a metabolic marker of K^+^ deficiency in plants and plays an important role in regulating the activity of vacuole channels (Bruggemann et al., 2002) It is also shown to be related to other stresses such as salinity and cold, as well as plant growth (Kou et al., 2018; Guo et al., 2019). Pro and Pro-derived metabolites were significantly decreased under K^+^ deficiency, and the expression of genes related to their regulation was altered accordingly (**Figures 2**, **10**; **Supplementary Tables 10, 11**). This is inconsistent with a previous finding, which reported that Pro and Pro-derived metabolites significantly accumulate under K^+^ deficiency (Xiong et al., 2022). This discrepancy can be attributed to the differences in Pro metabolism and synthesis in response to K^+^ deficiency in different plant species. GAD1 and spermine were enriched in the beta-alanine metabolism (Ko00,410) pathway, and both were down-regulated and positively correlated. SCL32, MYC2, SAUR36, NLP2, and L-anserine were enriched in the plant hormone signal transformation (Ko04075) pathway. SCL32, MYC2, and L-anserine were up-regulated, of which, SCL32 and MYC2 were positively correlated with L-Anserine; SAUR36 and NLP2 were down-regulated and negatively correlated with L-anserine, indicating that these genes regulate purine, beta-alanine metabolism, and plant hormone signal transduction. CSE, PHT1, PNC1, PER64, caffeic acid, and chlorogenic acid were enriched in the phenolproponoid biosynthesis (Ko00940) pathway; among these, CSE, PHT1, and PNC1 were down-regulated, whereas PER64, caffeic acid and chlorogenic acid were up-regulated, moreover, CSE and PHT1 were negatively correlated with caffeic acid and chlorogenic acid. PNC1 was also negatively correlated with chlorogenic acid, whereas PER64 was positively correlated with caffeic acid. Besides, chlorogenic acid, ALIS1, FLACCA, and biopterin were enriched in the folate biosynthesis pathway. Collectively, these results showed that CSE, PHT1, PNC1, PER64, ALIS1, and FLACCA regulate phenylpropanoid, flavonoid, stilbenoid, diarylheptanoid, and ginger biosynthesis (**Figure 10**; **Supplementary Table 10**).

## Conclusion

In this study, we compared the growth, nutrient contents, physiology, transcriptome, and metabolites of coconut seedlings under K^+^ deficiency and K^+^ sufficiency. The results showed that growth, nutrient contents, antioxidant enzyme activity, and endogenous hormone levels of coconut seedling leaves were affected by K^+^ deficiency stress. As K^+^ plays an important role in coconut growth, biomass, quality, yield, and disease resistance, it is essential to improve the K^+^ utilization efficiency of plants. The identification and further research on DEGs and DAMs under K^+^ deficiency would lay a foundation for improving K^+^ utilization efficiency of coconut plants in the future.

## Data availability statement

The datasets presented in this study can be found in online repositories. The name of the repository and accession number can be found below: NCBI; PRJNA914120.

## Author contributions

LL, YFY and YDY contributed to conception and design of the study. LL, SC and YDY organized the database. LL performed the statistical analysis. LL wrote the first draft of the manuscript. LL, SC, YW, WY, XY and YL wrote sections of the manuscript. All authors contributed to the article and approved the submitted version.
