# Supplementary material for: Integrated transcriptomic and metabolomic analyses reveal key metabolic pathways in response to potassium deficiency in coconut (Cocos nucifera L.) seedlings
Source: Front Plant Sci. 2023 Feb 13;14:1112264. doi: 10.3389/fpls.2023.1112264 (PMC9968814; doi:10.3389/fpls.2023.1112264)
Supplement: Supplementary file 1 [file DataSheet_1.docx]

Supplementary Material

Integrated Transcriptomic and Metabolomic Analyses Reveal Key Metabolic Pathways in Response to Potassium Deficiency in Coconut (*Cocos nucifera* L. ) Seedlings

Lilan Lu^1^*†, Siting Chen^1^†, Weibo Yang^1^†, Yi Wu^1^†, Yingying Liu^3^, XinXing Yin^1^, Yaodong Yang^1^*, Yanfang Yang^2^*

*** Correspondence:**

Lilan Lu: [lulilan1234@163.com](mailto:lulilan1234@163.com)

Yaodong Yang: yyang@catas.cn

Yanfang Yang: echoyyf@caf.ac.cn

†These authors have contributed equally to this work and share first authorship

## 1 Supplementary Data

## 1.1 Supplementary Figures

## Supplementary Figure 1. Gene expression analysis under K_0_ and K_ck_ treatments. (A) The gene expression distribution in K_0_ and K_ck_. (B) Principal component analysis (PCA) of expressed genes. (C) Each point represents a gene, and the X-axis represents the logarithm of the differential multiple of the expression of a gene in two samples; the Y-axis represents the negative logarithm of the statistical significance of the change in gene expression. The green dot represents the down-regulated differential expression gene, the red dot represents the up-regulated differential expression gene, and the black dot represents the non-differential expression gene.

## Supplementary Figure 2. Classification of GO annotation of differentially expressed genes (DEGs) in K_0_ vs. K_ck_. (A) GO annotation classification of up-regulated DEGs. (B) GO annotation classification of down-regulated DEGs. (C) GO annotation classification of all DEGs. The X-axis is the GO classification, the left side of Y-axis is the percentage of the gene number, and the right side of the Y-axis is the gene number.

## Supplementary Figure 3. GO enrichment network of DEGs from top pathways in K_0_ vs. K_ck_, including three categories. (A) Biological process of up-regulated DEGs. (B) Cellular component of up-regulated DEGs. (C) Molecular function of up-regulated DEGs. (D) Biological process of down-regulated DEGs. (E) Cellular component of down-regulated DEGs. (F) Molecular function of down-regulated DEGs. The color of the line represents different pathways, and the color of the gene node represents the multiple of differences. The larger the pathway node, the more genes enriched in the pathway.

## Supplementary Figure 4. Comparison of the log_2_fold change (K_0_/K_ck_) from 13 selected genes by RNA-seq and RT-qPCR in 3 biological replicates.

## Supplementary Figure 5. Top20 distribution histogram of up-regulated and down-regulated metabolites, with red indicating up-regulation and green indicating down-regulation.

## Supplementary Figure 6. Correlation analysis of DEGs and different accumulated metabolites(DAMs) in K_0_ and K_ck_. (A) Nine quadrant diagram.The X-axis is log_2_genes, and the Y-axis is log_2_metadata. The correlation between all genes and metabolites is calculated for each difference grouping based on the Pearson correlation method. Before calculating the correlation, the z-value transformation method is used for data preprocessing, and then the correlation coefficient (CC) and the P value of the correlation are used for screening. The screening threshold is CC > 0.80 and CCP < 0.05. (B) Hierarchical cluster heat map for correlation analysis of DEGs and DAMs. Each column is a sample of difference grouping, and each row represents a gene of significant difference or a metabolite of significant difference. Clustering significant difference metabolites or genes with differences in the same cluster have similar expression patterns. (C) Correlation coefficient matrix heat map of significantly DEGs and DAMs. Based on Pearson correlation analysis method, the correlation coefficient (CC) between significantly DEGs and DAMs was calculated. The correlation between significant DEGs and DAMs is shown in the form of correlation coefficient matrix heat map.The matrix diagram shows the correlation between significantly DEGs and DAMs. The correlation coefficient value r is between -1 and +1. The correlation coefficient r of genes and metabolites is expressed in color: r>0 indicates a positive correlation, which is expressed in red; r<0 indicates a negative correlation, expressed in blue. The darker the color, the stronger the correlation.

## Supplementary Figure 7. Transcription factor analysis in K_0_ vs. K_ck_. Square column blue indicates up-regulation and red indicates down-regulation

## 1.2 Supplementary Tables

## Supplementary Table 1 Primers used in qRT-PCR validation under K_ck_ and K_0_ treatments.

## Supplementary Table 2 The dry weight and plant height under under K_ck_ and K_0_ treatments.

**Supplementary Table 3** Mineral nutrients (% DW) in leaves of coconut under K_ck_ and K_0_ treatments. Data indicate means±SE (n = 3). Different letters behind the values in the same column for each tissue indicate significant differences between the treatments.

## Supplementary Table 4 Summary of the sequencing data generated for RNA-seq and mapping of the apple genome under K_ck_ and K_0_ treatments.Total Reads: Number of Clean Reads, calculated by single end; Mapped Reads: the number of Reads compared to the reference genome and the percentage in Clean Reads; Uniq Mapped Reads: the number of Reads compared to the unique position of the reference genome and the percentage in Clean Reads; Multiple Map Reads: the number of Reads compared to multiple locations of the reference genome and the percentage in Clean Reads.

## Supplementary Table 5 20910 genes with FPKM value in RNA-seq under K_0_ and K_ck_

## Supplementary Table 6 (A) 1003 DEGs in RNA-seq under K_0_ vs. K_ck._. (B) 884 DEGs annotation in RNA-seq in K_0_ vs. K_ck._

## Supplementary Table 7 (A) Analyses of Top 20 Gene ontology (GO) enrichment pathways in K_0_ vs. K_ck_.(B) Candidate genes related to top 20 Gene ontology (GO) enrichment pathways in K_0_ vs. K_ck_.

## Supplementary Table 8 (A) KEGG enrichment pathways based on 884 DEGs in K0 vs. Kck. (B) Candidate genes related to top 20 KEGG enrichment pathways based on 884 DEGs in K_0_ vs. K_ck._.

## Supplementary Table 9 164 different accumulated metabolites (DAMs) identified in coconut leaves in K_0_ vs. K_ck_.

## Supplementary Table 10 KEGG enrichment pathways of 28 different accumulated metabolites (DAMs) from 227 mtabolites K_0_ vs. K_ck._

## Supplementary Table 11 (A) KEGG enrichment pathways of correlations between genes and Metabolites in K_0_ vs. K_ck_. (B) Candidate genes related to 29 KEGG enrichment pathways of correlations between genes and Metabolites in K_0_ vs. K_ck_. in K_0_ vs. K_ck_.(132 DEGs, 100 DEGs in down-regulation, 32 DEGs in up regulation). (C) Candidate metabolites related to 29 KEGG enrichment pathways of correlations between genes and Metabolites in K_0_ vs. K_ck_. (21 DAMs, 13 DAMs in down-regulation, 8 DAMs in up-regulation).
