# Supplementary figures and images for: Integrated transcriptomic and metabolomic analyses reveal key metabolic pathways in response to potassium deficiency in coconut (Cocos nucifera L.) seedlings
Source: Front Plant Sci. 2023 Feb 13;14:1112264. doi: 10.3389/fpls.2023.1112264 (PMC9968814; doi:10.3389/fpls.2023.1112264)

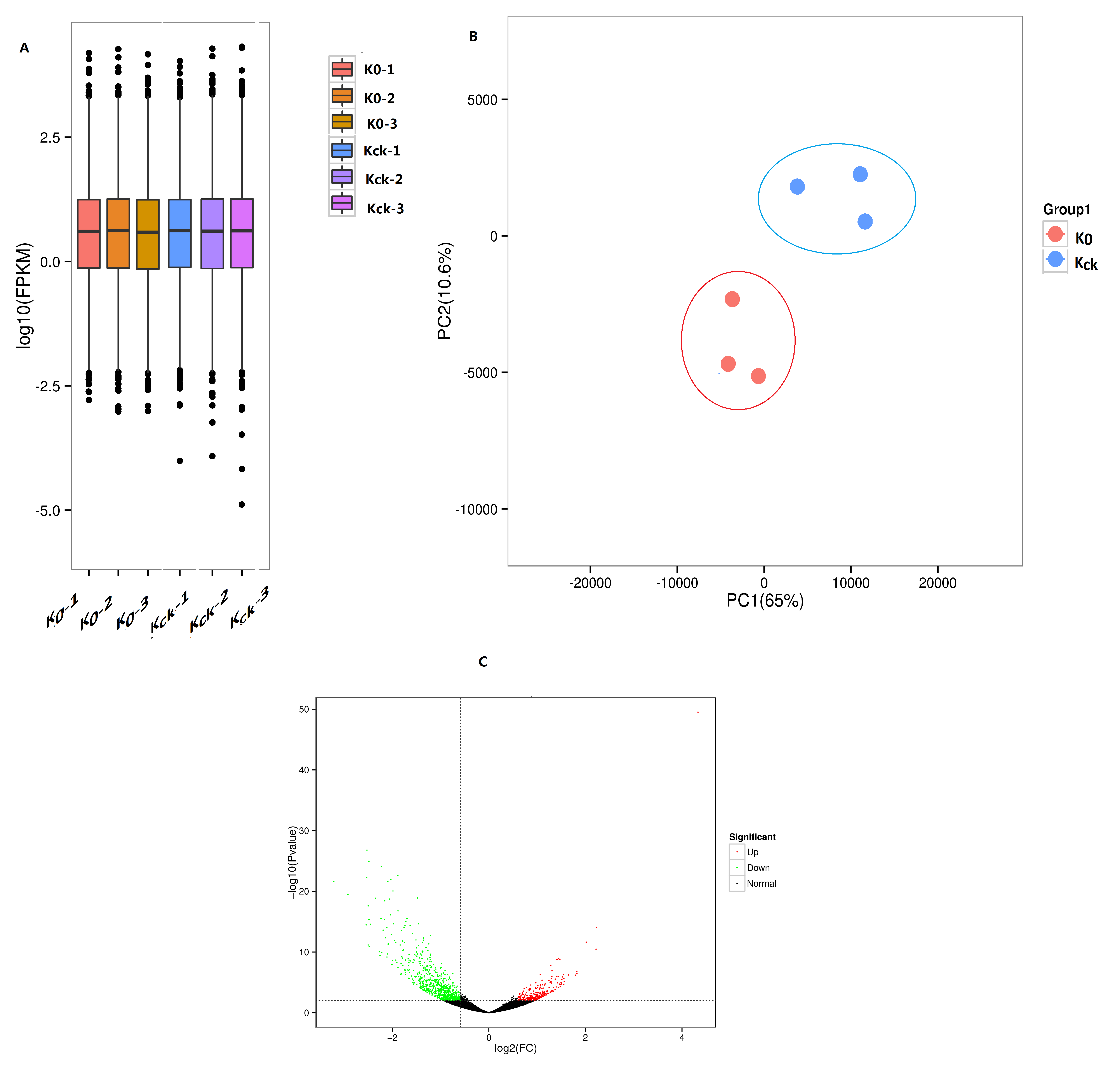

Supplement: Supplementary file 2 [file DataSheet_2.zip › Supplementary Figures 1-5/Fig S1.png]

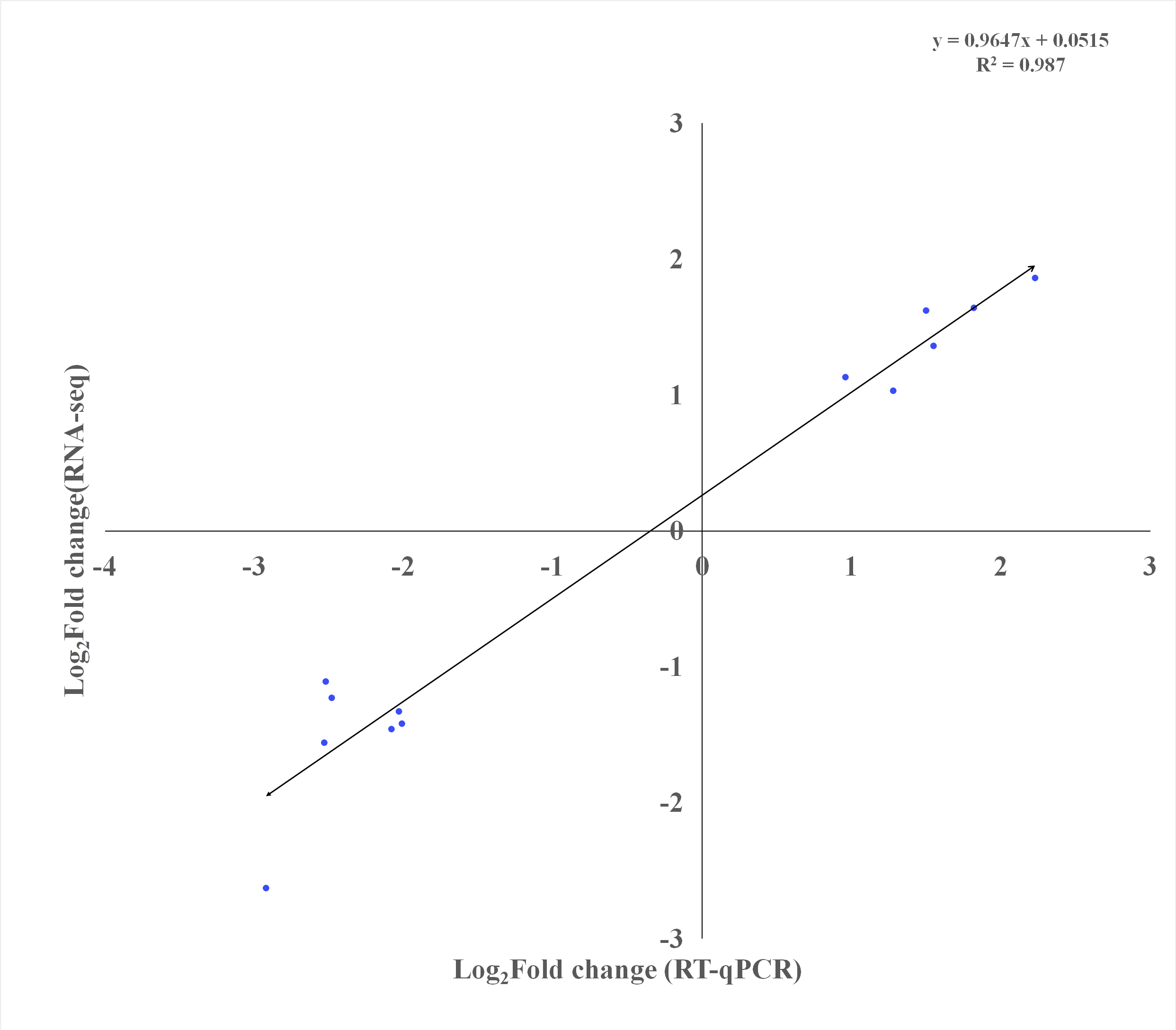

Supplement: Supplementary file 2 [file DataSheet_2.zip › Supplementary Figures 1-5/Fig.S4.dib]

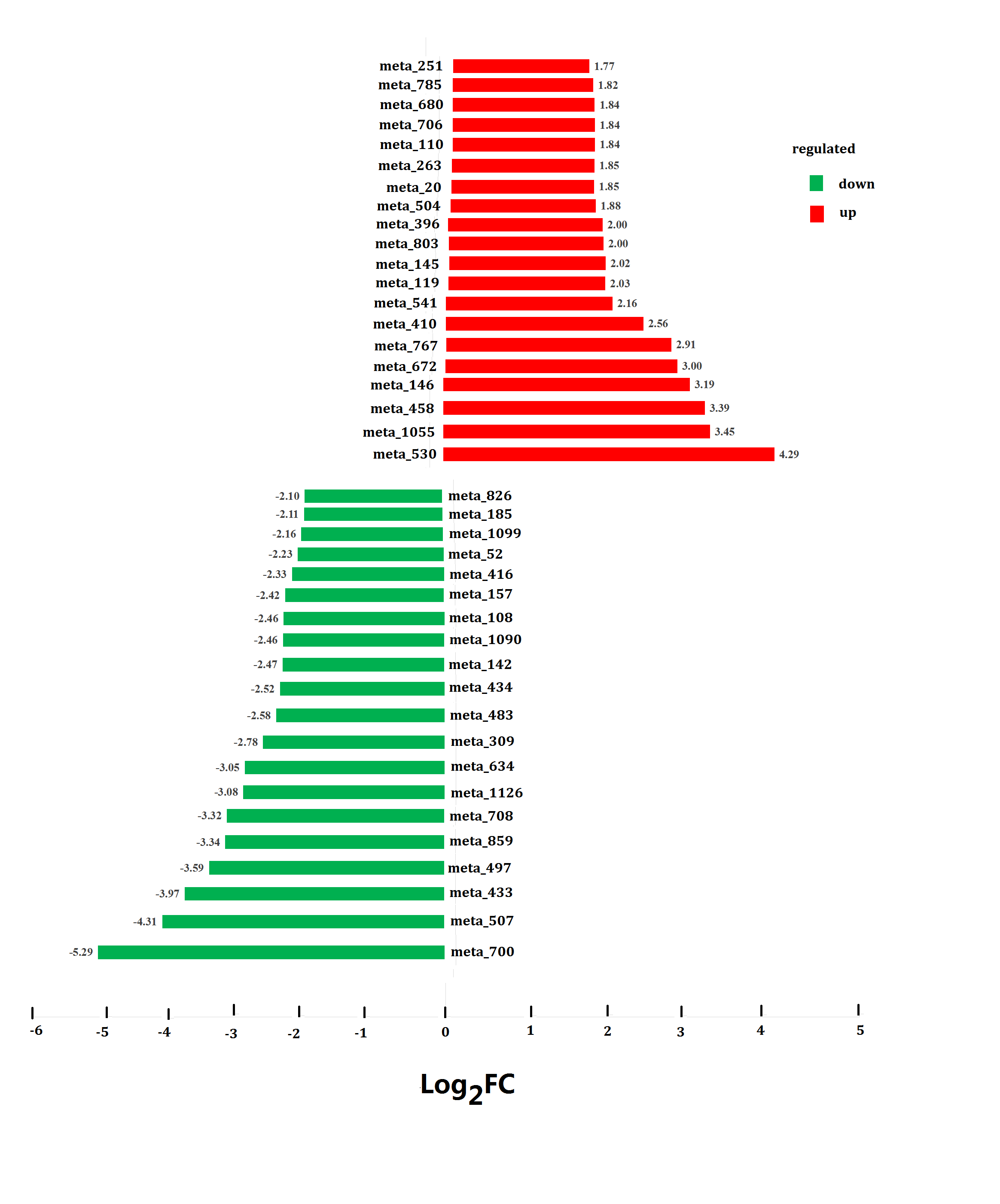

Supplement: Supplementary file 2 [file DataSheet_2.zip › Supplementary Figures 1-5/Fig.S5.tif]

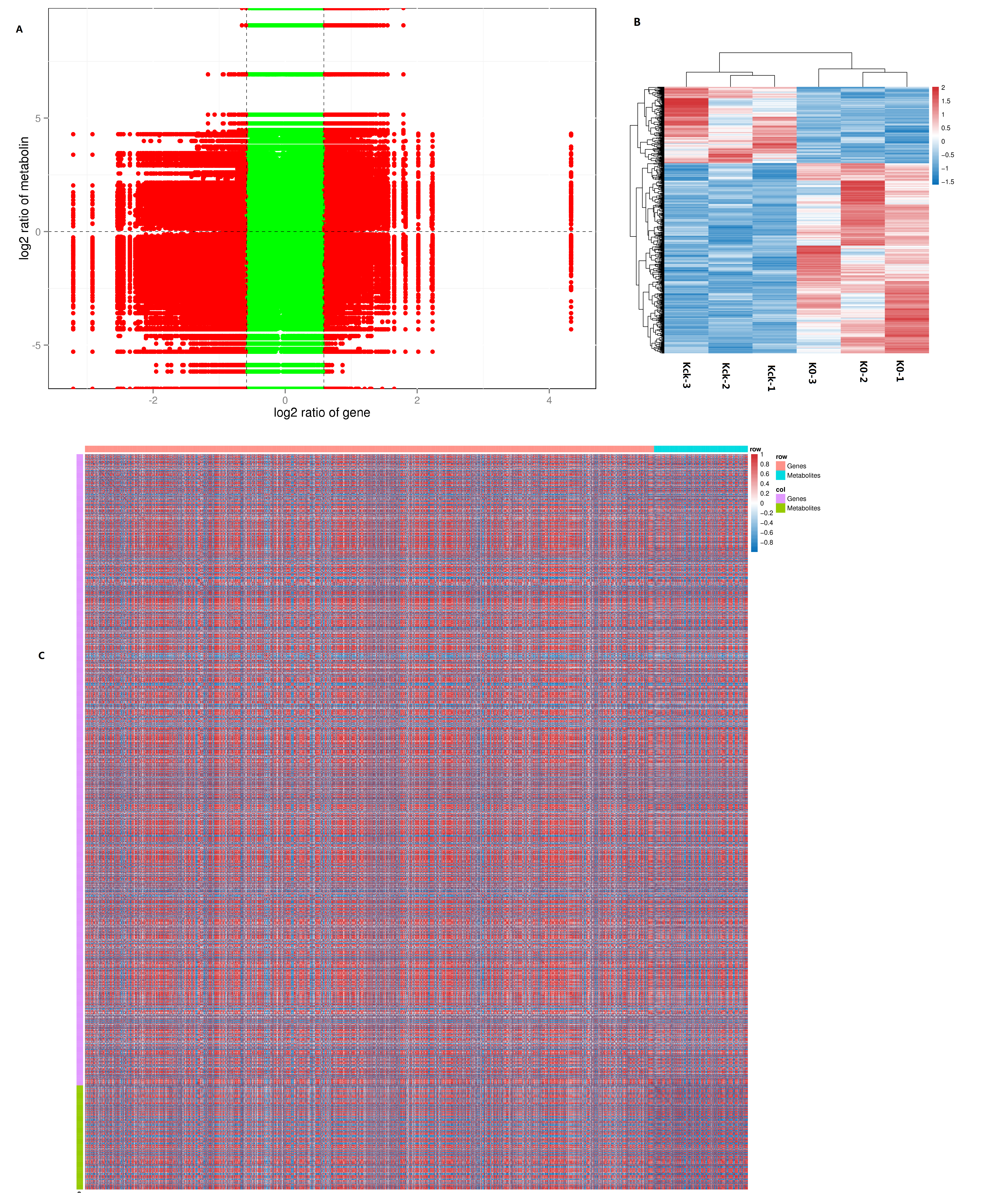

Supplement: Supplementary file 3 [file DataSheet_3.zip › Supplementary Figures6-7 and Tables 1-11/Fig.S6.png]

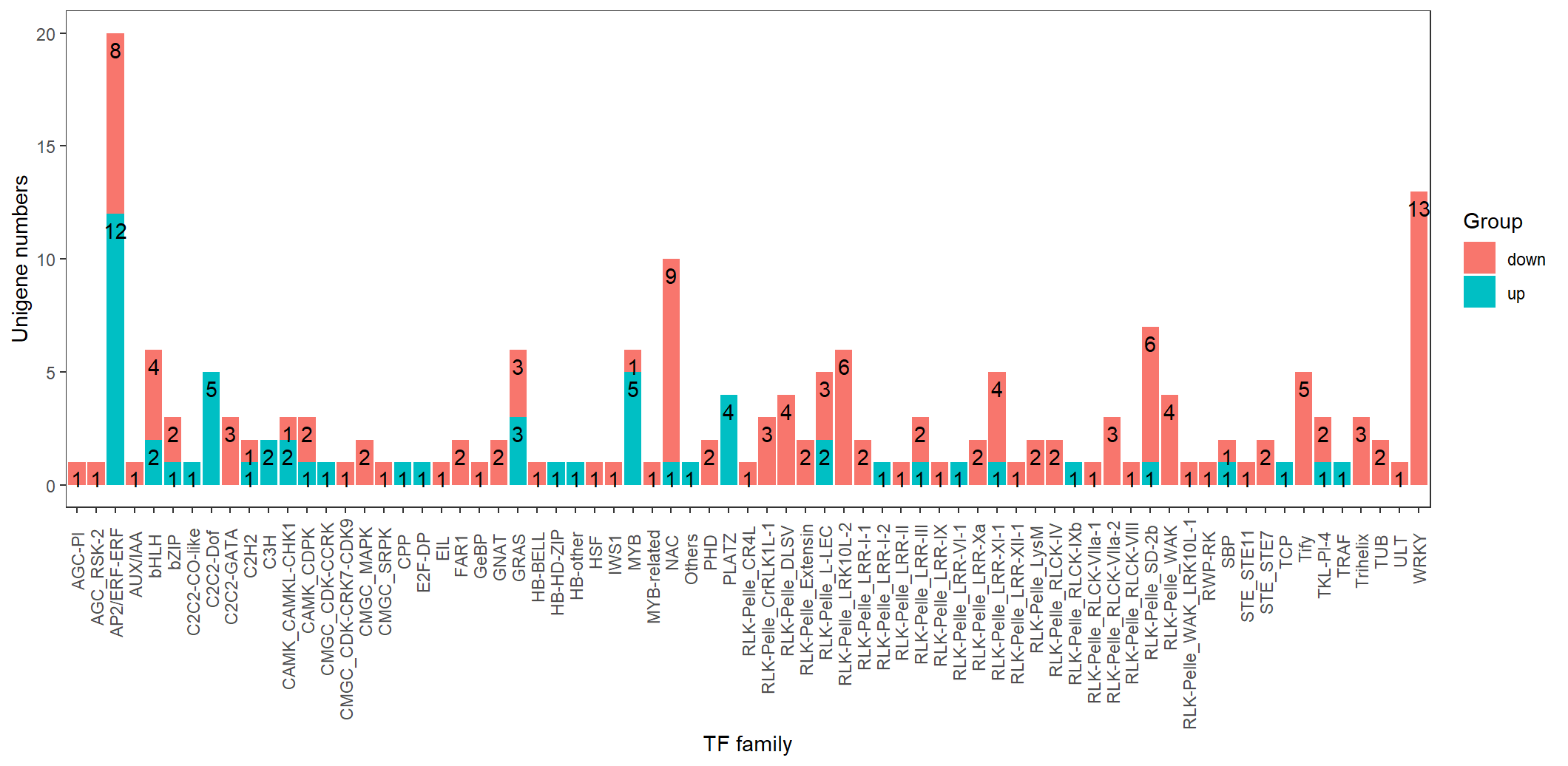

Supplement: Supplementary file 3 [file DataSheet_3.zip › Supplementary Figures6-7 and Tables 1-11/Fig.S7.png]
