# Supplementary material for: Integrated transcriptomic and metabolomic analyses reveal key metabolic pathways in response to potassium deficiency in coconut (Cocos nucifera L.) seedlings
Source: Front Plant Sci. 2023 Feb 13;14:1112264. doi: 10.3389/fpls.2023.1112264 (PMC9968814; doi:10.3389/fpls.2023.1112264)
Supplement: Supplementary file 3 [file DataSheet_3.zip › Supplementary Figures6-7 and Tables 1-11/SupplementaryTables 1-4.docx]

**Table S1.** Primers used in qRT-PCR validation under K_ck_ and K_0_ treatments.

|  |  |  |  |
| --- | --- | --- | --- |
| **Gene ID** | **Gene Symbol** | **Forward primer (5->3)** | **Reverse primer (5->3)** |
| β-actin | β-actin | TCCAATCTATGAGGGATACACGC | TCTTCATTAGATTATCCGTGAGGTC |
| COCN_GLEAN_10008378 | CIPK14 | GATCCTCTACGTCCTCAA | GGTAGATCTTGCGGTACA |
| CUFF17.34.1 | HAK8 | GCACTTGGTGGCAATCTT | TGGAAGGTTGGTGACGAA |
| COCN_GLEAN_10020942 | CIPK25 | AGCCCCGCCAGCAGTGTC | TATATGATTGACCGACGC |
| COCN_GLEAN_10005263 | CML46 | ATGGGTCCCGTGGTGGCT | GAGGACAGCTTCTGCTGA |
| COCN_GLEAN_10010696 | CAB91R | ATGGCTGCCACGATGGCC | TTTGTCCCCGGCAAGTGA |
| COCN_GLEAN_10008284 | SAUR71 | ATGGGTTGGAGAAGAAAG | ATCTCAAAAGATAGATGA |
| COCN_GLEAN_10007191 | ERF026 | ATGGCTGCCAACCCTCCG | CTGTGGAGCTACACTTAA |
| COCN_GLEAN_10007380 | LECRK91 | ATGGTTCCCTCCGGTTCG | CATAGATCTGACATGTAA |
| COCN_GLEAN_10020974 | PHT1 | ATTTCGCGACCACTGGCC | CCCCCACTCTAGCACGCA |
| COCN_GLEAN_10008971 | WRKY41 | ATGGAGAAGGGGACAAG | TCCGACTTTTTCCAATAA |
| COCN_GLEAN_10013289 | RBOHC | ATGCAGAAGATGGGGAC | CACAAGGAGAACTTCTAG |
| COCN_GLEAN_10021958 | ABCG39 | ATGCGGGGAAGCATGCG | AACTTCCAGAGAAGATGA |
| COCN_GLEAN_10022669 | WAK2 | ATGAGAAGTAGAACAGC | TTCAATATCATGAGATGA |

**Table S2.** The dry weight and plant height under under K_ck_ and K_0_ treatments.

| Treatment | Plant height（cm） | Dry weight(g/plant)  M+SD |
| --- | --- | --- |
| K_ck_ | 112.70±2.36 a | 163.67±0.42 a |
| K_0_ | 88.10±1.56 b | 120.38±0.16 b |

**Table S3.** Mineral nutrients (% DW) in leaves of coconut under K_ck_ and K_0_ treatments. Data indicate means ± SDs (n=3). Different letters behind the values in the same column for each tissue indicate significant differences between the treatments.

| Treatment | The element uptake of leaves (%) | | |
| --- | --- | --- | --- |
|  | K | N | P |
| K_ck_ | 1.22±0.091a | 1.86±0.093 a | 0.111±0.006 a |
| K_0_ | 0.63±0.023 b | 1.48±0.074 a | 0.101±0.002 a |

**Table S4.** Summary of the sequencing data generated for RNA-seq and mapping of the apple genome under K_ck_ and K_0_ treatments.Total Reads: Number of Clean Reads, calculated by single end. Mapped Reads: the number of Reads compared to the reference genome and the percentage in Clean Reads. Uniq Mapped Reads: the number of Reads compared to the unique position of the reference genome and the percentage in Clean Reads. Multiple Map Reads: the number of Reads compared to multiple locations of the reference genome and the percentage in Clean Reads.

| Sample | Total Reads | Mapped Reads | Uniq Mapped Reads | Multiple Map Reads |
| --- | --- | --- | --- | --- |
| K_ck-1_ | 42732396 | 40,478,526 (94.73%) | 38,260,101 (89.53%) | 2,218,425 (5.19%) |
| K_ck-2_ | 41367808 | 39,245,014 (94.87%) | 37,146,523 (89.80%) | 2,098,491 (5.07%) |
| K_ck-3_ | 41718396 | 39,509,467 (94.71%) | 37,380,434 (89.60%) | 2,129,033 (5.10%) |
| K_0-1_ | 39896122 | 37,889,585 (94.97%) | 35,974,425 (90.17%) | 1,915,160 (4.80%) |
| K_0-2_ | 41228320 | 39,112,075 (94.87%) | 37,015,736 (89.78%) | 2,096,339 (5.08%) |
| K_0-3_ | 43590868 | 41,240,095 (94.61%) | 38,890,800 (89.22%) | 2,349,295 (5.39%) |
